# Relationship Estimation from Whole-Genome Sequence Data

**DOI:** 10.1371/journal.pgen.1004144

**Published:** 2014-01-30

**Authors:** Hong Li, Gustavo Glusman, Hao Hu, Shankaracharya, Juan Caballero, Robert Hubley, David Witherspoon, Stephen L. Guthery, Denise E. Mauldin, Lynn B. Jorde, Leroy Hood, Jared C. Roach, Chad D. Huff

**Affiliations:** 1 Institute for Systems Biology, Seattle, Washington, United States of America; 2 Department of Epidemiology, The University of Texas MD Anderson Cancer Center, Houston, Texas, United States of America; 3 Department of Human Genetics, University of Utah School of Medicine, Salt Lake City, Utah, United States of America; 4 Department of Pediatrics, University of Utah School of Medicine, Salt Lake City, Utah, United States of America; The University of Queensland, Australia

## Abstract

The determination of the relationship between a pair of individuals is a fundamental application of genetics. Previously, we and others have demonstrated that identity-by-descent (IBD) information generated from high-density single-nucleotide polymorphism (SNP) data can greatly improve the power and accuracy of genetic relationship detection. Whole-genome sequencing (WGS) marks the final step in increasing genetic marker density by assaying all single-nucleotide variants (SNVs), and thus has the potential to further improve relationship detection by enabling more accurate detection of IBD segments and more precise resolution of IBD segment boundaries. However, WGS introduces new complexities that must be addressed in order to achieve these improvements in relationship detection. To evaluate these complexities, we estimated genetic relationships from WGS data for 1490 known pairwise relationships among 258 individuals in 30 families along with 46 population samples as controls. We identified several genomic regions with excess pairwise IBD in both the pedigree and control datasets using three established IBD methods: GERMLINE, fastIBD, and ISCA. These spurious IBD segments produced a 10-fold increase in the rate of detected false-positive relationships among controls compared to high-density microarray datasets. To address this issue, we developed a new method to identify and mask genomic regions with excess IBD. This method, implemented in ERSA 2.0, fully resolved the inflated cryptic relationship detection rates while improving relationship estimation accuracy. ERSA 2.0 detected all 1^st^ through 6^th^ degree relationships, and 55% of 9^th^ through 11^th^ degree relationships in the 30 families. We estimate that WGS data provides a 5% to 15% increase in relationship detection power relative to high-density microarray data for distant relationships. Our results identify regions of the genome that are highly problematic for IBD mapping and introduce new software to accurately detect 1^st^ through 9^th^ degree relationships from whole-genome sequence data.

## Author Summary

The determination of the relationship between a pair of individuals is a fundamental application of genetics. The most accurate methods for relationship estimation rely on precise, localized estimates of genetic sharing between individuals. Earlier methods have generated these estimates from high-density genetic marker data. We performed relationship estimation using whole-genome sequence data for 1490 known pairwise relationships among 258 individuals in 30 families along with 46 population samples as controls. Our results demonstrate that complexities specific to whole-genome sequencing result in regions of the genome that are prone to false-positive estimates of genetic sharing. We provide a map of these spurious IBD regions and introduce new methods, implemented in the software package ERSA 2.0, to control for spurious IBD. We show that ERSA 2.0 provides a 5% to 15% increase in relationship detection power for distant relationships with whole-genome sequence data relative to high-density genetic marker data.

## Introduction

The identification of related individuals from genetic data has a broad range of applications. The validation of known relationships in familial disease-gene studies ensures that pedigree errors or sample switches do not adversely affect power [1]. In case-control studies, the removal of related individuals is a standard quality control step to avoid spurious associations [2]. Population genetics studies typically must either explicitly account for familial relationships [3], or else exclude related individuals from analyses that rely on random mating and representative sampling assumptions [4]. Genetic relationship identification is also widely used in a number of forensic applications, including criminal investigations, identification of missing persons and victims of mass disasters [5], [6].

Methods applicable to the detection of close relationships have been available for decades [1], [7]. These methods typically rely on either genome-wide estimates of identity-by-descent (IBD) [8] or joint inference of IBD and relationships using sparse genetic markers [9]. With approximately 1,000 highly polymorphic markers, such methods are well powered to accurately identify relationships as distant as 3^rd^-degree relatives [9], but these methods do not benefit from further increases in marker density [10]. With the introduction of single-nucleotide polymorphism (SNP) microarrays, increased marker density enabled the accurate detection of local IBD segments. Newer relationship estimation methods take advantage of local IBD segment data to increase the range of detectable relationships [10], [11]. The relationship estimation software that we previously developed, Estimation of Recent Shared Ancestry (ERSA), has high power to detect relationships as distant as 8^th^-degree relatives (e.g., 3^rd^ cousins once removed) from high-density SNP microarray data [10].

Whole-genome sequence (WGS) should represent the final step in increasing marker density, and thus, improved relationship detection accuracy. However, with the current complexity of WGS, which is based on high-throughput short-reads mapped to a legacy reference genome, a number of technical challenges must be overcome before potential improvements in relationship detection accuracy can be realized. To assess these challenges, we analyzed WGS data for 1490 distinct pairwise relationships from 258 individuals in 30 families (see Table 1). Our results highlight new issues specific to relationship estimation from WGS data and introduce new methods in ERSA 2.0 to mitigate these issues.

**Table 1:** Description of sequenced families.

Family number	Number of sequenced family members	Number of pairwise relationships	Most distant relationships	Inferred population
1	16	120	5	ASI
2	7	21	2	ASI
3	25	300	3	CEU
4	10	45	3	CEU
5	4	6	1	CEU
6	5	10	1	CEU
7	5	10	1	CEU
8	7	21	12	CEU
9	10	45	11	CEU
10	4	6	6	CEU
11	17	136	2	CEU
12	4	6	1	CEU
13	4	6	1	CEU
14	5	10	1	CEU
15	4	6	2	CEU
16	15	105	4	CEU
17	10	45	3	CEU
18	7	21	2	CEU
19	7	21	2	CEU
20	8	28	3	CEU
21	9	36	2	CEU
22	15	105	3	CEU
23	4	6	1	CEU
24	4	6	5	CEU
25	4	6	1	CEU
26	4	6	1	CEU
27	4	6	1	CEU
28	6	15	2	MXL
29	25	300	5	MXL
30	9	36	1	MXL
Total	258	1490		

## Results

**Figure 1:**
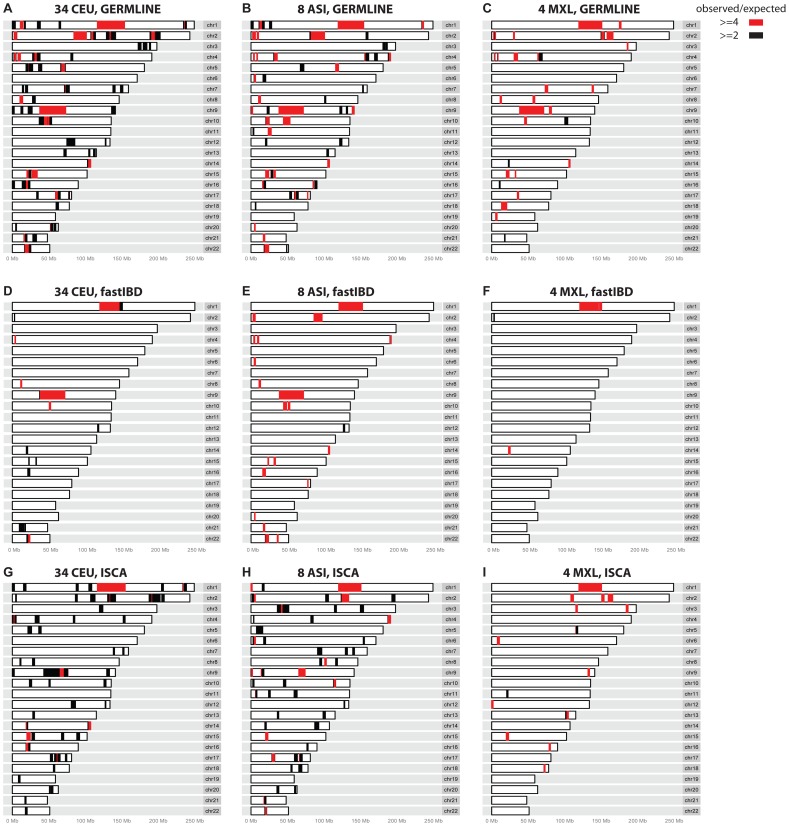
Regions where excess IBD is detected by three IBD methods among the control populations. Regions that give rise to excess IBD inferences in GERMLINE (A–C), fastIBD (D–F), and ISCA (G–I) IBD. Black and red shading denotes degree of excess IBD detected (see legend).

To evaluate relationship-estimation accuracy on WGS data, we first inferred IBD segments between each pair of individuals with three different methods: Genetic Error-tolerant Regional Matching with Linear-time Extension (GERMLINE), Beagle's fastIBD, and Inheritance State Consistency Analysis (ISCA) [12]–[14]. We then applied ERSA separately to each of the three resulting IBD-segment datasets. For our initial analysis of control genomes from putatively unrelated individuals of European ancestry, we set the chance of falsely detecting a relationship between unrelated individuals to 0.1% (α = 0.001). With this threshold, we detected a significant relationship of 9^th^-degree or closer using GERMLINE in approximately 10% of all pairs of individuals. The estimated level of cryptic relatedness was 10-fold higher than we previously observed from high-density microarray data in this population [10], and thus was a strong indication of an elevated false-positive rate (Table 2). After further investigation, we identified several regions of the genome that were detected to be IBD far more often than would be expected by chance among pairs of controls (see Materials and Methods). Table 3 shows 14 regions of the genome greater than 5 cM with detected pairwise IBD identified in GERMLINE that exceeds the expected pairwise IBD by at least 4-fold between European controls. The regions of spurious IBD were largely consistent between the three IBD methods and among European, East Asian, and Mexican American populations (Figures 1 and S7, Tables 3 and S2, S3), which is a strong indication that the IBD segments in these regions are artifactual. To account for these spurious IBD segments, we developed a procedure within ERSA 2.0 to identify and mask regions of the genome with excess IBD in controls (see Materials and Methods). After applying this procedure, the rate of detected relationships among the European controls decreased from 10% to 1% at α = 0.001 using ERSA 2.0 and GERMLINE, which is the rate of cryptic relationships that we previously observed in this population [10]. In addition to region masking, we also implemented new models in ERSA 2.0 that improve the accuracy of relationship estimates for closely related individuals (see Materials and Methods). Although fastIBD detected many of the same regions as GERMLINE and ISCA, the rate of spurious IBD detection was generally much lower (Table 1 and Figure 1). For this reason, the rate of detected relationships among European controls was less than 0.002 at α = 0.001 using ERSA 2.0 and fastIBD, even without masking spurious IBD segments.

**Table 2:** Predicted relationships for 595 individual pairs in three groups of population controls: 561 pairs from 34 European controls (CEU), 28 pairs from 8 East Asian controls (ASI), and 6 pairs from 4 Mexican-American controls (MXL).

Predicted relationship degree	GERMLINE, before masking	GERMLINE, after masking	ISCA, before masking	ISCA, after masking	fastIBD, before masking	fastIBD, after masking
**All populations (595 pairs)**						
5	0 (0, 1)	0 (0, 0)	0 (0, 0)	0 (0, 0)	0 (0, 0)	0 (0, 0)
6	3 (0, 9)	0 (0, 1)	2 (0, 0)	0 (0, 0)	0 (0, 0)	0 (0, 0)
7	33 (0, 28)	0 (0, 0)	74 (0, 0)	0 (0, 0)	0 (0, 0)	0 (0, 0)
8	54 (0, 20)	2 (0, 0)	171 (0, 0)	2 (0, 0)	0 (0, 0)	0 (0, 0)
9	14 (1, 7)	5 (1, 0)	29 (2, 2)	1 (2, 2)	1 (0, 4)	0 (0, 3)
Unrelated	491 (594, 530)	588 (594, 594)	319 (593, 593)	592 (593, 593)	594 (595, 591)	595 (595, 592)
**CEU only (561 pairs)**						
6	0 (0, 0)	0 (0, 0)	2 (0, 0)	0 (0, 0)	0 (0, 0)	0 (0, 0)
7	19 (0, 21)	0 (0, 0)	67 (0, 0)	0 (0, 0)	0 (0, 0)	0 (0, 0)
8	53 (0, 20)	2 (0, 0)	161 (0, 0)	2 (0, 0)	0 (0, 0)	0 (0, 0)
9	14 (1, 7)	5 (1, 0)	29 (2, 2)	1 (2, 2)	0 (0, 4)	0 (0, 3)
Unrelated	475 (560, 513)	554 (560, 561)	302 (559, 559)	558 (559, 559)	561 (561, 557)	561 (561, 558)
**ASI only (28 pairs)**						
5	0 (0, 1)	0 (0, 0)	0 (0, 0)	0 (0, 0)	0 (0, 0)	0 (0, 0)
6	3 (0, 9)	0 (0, 1)	0 (0, 0)	0 (0, 0)	0 (0, 0)	0 (0, 0)
7	14 (0, 1)	0 (0, 0)	6 (0, 0)	0 (0, 0)	0 (0, 0)	0 (0, 0)
8	1 (0, 0)	0 (0, 0)	10 (0, 0)	0 (0, 0)	0 (0, 0)	0 (0, 0)
9	0 (0, 0)	0 (0, 0)	0 (0, 0)	0 (0, 0)	1 (0, 0)	0 (0, 0)
Unrelated	10 (28, 17)	28 (28, 27)	12 (28, 28)	28 (28, 28)	27 (28, 28)	28 (28, 28)
**MXL only (6 pairs)**						
7	0 (0, 0)	0 (0, 0)	1 (0, 0)	0 (0, 0)	0 (0, 0)	0 (0, 0)
Unrelated	6 (6, 6)	6 (6, 6)	5 (6, 6)	6 (6, 6)	6 (0, 0)	6 (0, 0)

Numerical values in the table are results of WGS data, numerical values in parentheses are results of “SNP microarray” and “exon” data.

**Table 3:** Genomic Regions in hg19 coordinates of at least 5-to-expected IBD of at least 4-fold.

				Ratio of observed to expected IBD				
Chromosome	Starting position	Ending position	Genetic length (in cM)	GERMLINE Europe	GERMLINE Asia	ISCA Europe	fastIBDEurope	GERMLINE Europe Affy 6.0
**chr9**	**38,293,483**	**72,605,261**	8.15	39	13	10	2	4
**chr8**	**10,428,647**	**13,469,693**	7.96	38	26	2	4	3
**chr21**	**16,344,186**	**19,375,168**	6.91	22	15	2	0	2
**chr10**	**44,555,093**	**53,240,188**	7.58	22	21	2	3	2
**chr22**	**16,051,881**	**25,095,451**	20.82	22	22	3	6	1
**chr2**	**85,304,243**	**99,558,013**	6.53	21	21	2	1	4
**chr1**	**118,434,520**	**153,401,108**	9.95	19	33	47	811	9
**chr15**	**20,060,673**	**25,145,260**	10.46	15	20	42	0	11
**chr17**	**77,186,666**	**78,417,478**	5.66	11	7	0.1	0	0.3
**chr15**	**27,115,823**	**30,295,750**	9.29	9	3	3	0	15
**chr17**	**59,518,083**	**64,970,531**	6.23	9	4	4	0	3
**chr2**	**132,695,025**	**141,442,636**	9.16	7	0	4	0	10
**chr16**	**19,393,068**	**24,031,556**	6.18	6	2	5	0	1
**chr2**	**192,352,906**	**198,110,229**	5.04	4	2	4	0	12
**Total**	**14 regions**		119.92					

**Figure 2:**
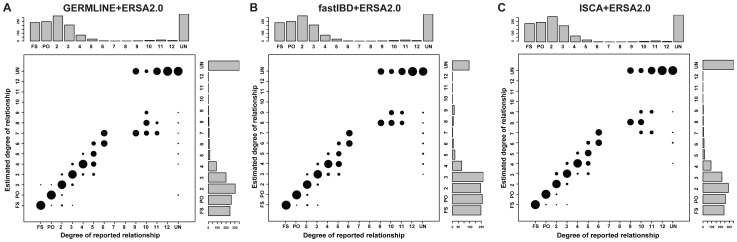
Performance of relationship estimation in 30 sequenced families using (A) GERMLINE-ERSA2.0, (B) fastIBD-ERSA2.0, and (C) ISCA-ERSA2.0. Area of the circles indicates the percentage of individual pairs whose estimated degrees of relationship are exactly the same as reported relationship. FS: full sibling. PO: parent offspring. UN: unrelated individuals. All ERSA analyses employed IBD masking. Histograms represent the number of pairs in each relationship category. Most of the pedigrees were ascertained on the basis of common, complex or rare, Mendelian diseases. As we have previously reported, this ascertainment can produce a downward bias in distant relationship estimates [10], which may account for the differences in relationship estimates between sequenced and simulated pedigrees for 10^th^ through 12^th^ degree relationships (see Figure S5).

Figure 2 summarizes the ERSA 2.0 results from the 30 pedigrees (see also Table S1 and Figure S3). ERSA 2.0 detected all 1^st^ through 6^th^ degree relationships and 55% of 9^th^ through 11^th^ degree relationships in the 30 pedigrees. The performance of ERSA was very similar across the three IBD detection methods, with approximately a 5% difference in exact relationship prediction accuracy.

**Figure 3:**
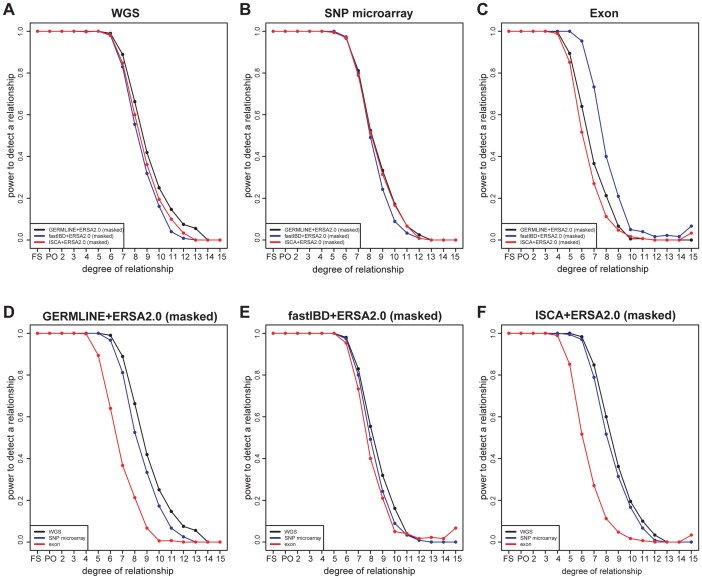
Power of relationship estimation for simulated pedigrees using different methods and markers. (A–C) Comparison of three methods: GERMLINE-ERSA2.0, fastIBD-ERSA2.0, and ISCA-ERSA2.0. (A) “WGS” represents simulated whole-genome data, (B) “SNP microarray” represents Affymetrix 6.0 microarray data, and (C) “exon” represents whole-exome data. (D–F) Comparison of different marker set: “WGS”, “SNP microarray”, and “exon”. (D) GERMLINE-ERSA2.0, (E) fastIBD-ERSA2.0, and (F) ISCA-ERSA2.0.

Although the 30 pedigrees included 1490 documented pairwise relationships, only 28 of these relationships were more distant than 6^th^ degree. To evaluate performance of ERSA 2.0 and IBD detection methods for more-distant relationships, we simulated WGS data in 15-generation pedigrees (See Materials and Methods; Figures S1). ERSA 2.0 performed well with all three IBD detection methods (Figures 3, S5, and S10). For each method, we observed greater than 95% power to detect relationships as distant as 5^th^ degree and greater than 50% power to identify relationships as distant as 8^th^ degree (*α* = 0.001). We also performed IBD estimation using subsets of the data to represent SNP microarray data (using the set of positions from the Affymetrix 6.0 array) and whole-exome data. The increase in marker density from SNP microarray data to WGS data resulted in a 5% to 15% increase in power for distant relationships between 7^th^ and 11^th^ degree (Figure 3). Restricting markers to exonic regions reduced power relative to WGS data, with a 10% to 60% decrease in power for GERMLINE-ERSA and ISCA-ERSA and a 5% to 10% decrease in power for fastIBD-ERSA with 5^th^ through 12^th^ degree relationships (Figure 3). With exonic markers, we observed a modest increase in the rate of detected relationships among control populations of between 0.2 to 0.5% (Table 2). However, for exonic markers in simulated families, the power to detect distant relationships (10^th^–15^th^ degree) increased by as much as 5%. This increase in power is very likely to be an artifact and is probably an indication that the increased difficulty of detecting IBD data from exonic markers may lead to improperly calibrated Type I error in ERSA 2.0 for some whole-exome datasets.

To compare ERSA to an approach that does not rely on local IBD segment estimates, we also estimated pairwise relationships using RELPAIR, a method that jointly estimates IBD and relationships using sparse marker data. RELPAIR's performance was similar to ERSA for 1^st^ and 2^nd^ degree relationships. Both approaches accurately differentiate between parent-offspring and full-sibling relationships in over 96% of comparisons. RELPAIR had no ability to differentiate between 3^rd^ through 5^th^ degree relationships and had low power to detect relationships more distant than 5^th^ degree (Figures S4 and S6), as previously reported [10].

## Discussion

Our results demonstrate that several regions of the genome exhibit an excess of detected IBD with state-of-the-art WGS and IBD detection methods. These suspect IBD regions were typically characterized by an increase in departures from Hardy-Weinberg Equilibrium and were often near centromeric regions. Gaps in the recombination map and human reference assembly were also overrepresented. For example, although the regions in Table 3 represent less than 5% of the human genome, they represent 13% of the centromeric regions and 47% of the unmappable heterochromatic regions of the genome (“Gap” tracks in the UCSC Genome Browser). Notably, the IBD regions were not enriched for repetitive segments of the genome [15]. Because many of the regions were identified using three distinct IBD detection methods, the regions we identified with spurious IBD are unlikely to be the result of IBD detection algorithm errors. Although strong recent positive selection can produce this effect on a population scale, positive selection is unlikely to explain this result because the regions we identified were typically detected among Europeans, East Asians, and Mexican Americans and were far larger than any previous reported genomic signal of positive selection in humans (Figure 1 and Table 3). In addition, we observed very little overlap between the regions identified in Table 3 and a genome-wide search for genomic regions influenced by positive selection based on signals of excess IBD (Table S4) [16]. The regions identified using WGS data usually exhibited excess IBD in Affymetrix high-density microarray data as well, although at lower magnitudes and with smaller segment sizes (Table 3), suggesting that the excess IBD is not simply due to artifacts specific to high-throughput short-read resequencing. One potential explanation is that errors in published genetic maps in these regions overestimate the size of the IBD segments when measured by genetic distance. This hypothesis is supported by the gaps in the published recombination maps and relatively sparse high-density microarray marker density in these regions. Gaps in the human reference assembly may be another contributing factor, both directly due to the absence of markers and indirectly as a general indicator of mapping difficulty in flanking regions. The increased rate of deviations from Hardy-Weinberg equilibrium could also provide a partial explanation, given that erroneous heterozygote calls can result in false inferences of IBD segments. Some of the regions we have identified may be the result of long-range haplotypes with limited recombination between haplotypes [17], [18]. Of the 14 regions identified in Table 3, seven overlapped with regions previously identified in studies of long-range linkage disequilibrium (Tables S5, S6) [17], [18]. One of these regions, at position 10.5 to 13.5 Mb on chromosome 8, overlaps with a known inversion polymorphism that suppresses recombination between haplotypes [19].

**Figure 4:**
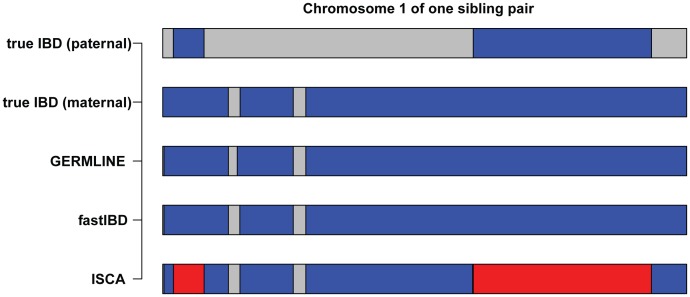
Comparison of IBD inferred by GERMLINE, fastIBD, and ISCA. IBD between one simulated sibling pair was shown as an example (chromosome 1). Blue segments indicate haploid-identity (IBD1) and red segments indicate diploid-identity (IBD2).

Our analysis focused on three complementary pairwise IBD detection methods, GERMLINE, fastIBD, and ISCA (Figure 4). GERMLINE accepts phased genotype data and employs a haplotype hashing algorithm to reduce computation time [12]. Although GERMLINE is capable of analyzing unphased data, in our experience IBD segment identification and subsequent relationship estimation accuracy are both greatly reduced. Beagle fastIBD employs a similar approach to GERMLINE, but obtains multiple estimates of haplotype phase internally and evaluates each of these haplotypes [13]. The rate of spurious IBD detection in fastIBD was substantially lower than GERMLINE and ISCA, and we did not observe an excess of detected relationships among control populations with fastIBD and ERSA, even in the absence of masking (Table 2). However, the power of ERSA 2.0 to detect relationships was slightly reduced with fastIBD relative to the other two methods (Figure 3). Both GERMLINE and fastIBD are well optimized for large sample sizes, but neither distinguishes between haploid-identical regions (IBD1) and diploid-identical regions (IBD2). We originally described ISCA as a method for simultaneous detection of all blocks of identity throughout a pedigree [20], [21]. ISCA also performs well for detecting both IBD1 and IBD2 segments between pairs of individuals with an unknown relationship. ISCA employs a Hidden Markov Model that identifies both IBD1 and IBD2 segments [20]. Because ISCA is optimized for whole-genome data, the algorithm suppresses noise from segments of the genome that give rise to false positive IBD1 and IBD2 regions, such as compressions, centromeres, hemizygous regions, CNVs, reference gaps, and other irregularities [20]. Unlike GERMLINE and fastIBD, ISCA does not require phased data or population controls. However, because ISCA's execution time scales linearly with the number of individual pairs, it is slower than both GERMLINE and fastIBD for large sample sizes.

All of the datasets we evaluated included complete documentation of missing genotypes (i.e. no-calls). In our experience, missing genotype data are essential to accurate IBD estimation. Variant call data that does not report missing genotypes should not be used for relationship estimation.

WGS data present new challenges for IBD detection and relationship estimation. Using existing approaches, we observed a major increase in the detection of spurious IBD segments and false-positive relationships from WGS data of population controls. We provide a map of spurious IBD regions in the human reference sequence and present methods implemented in ERSA 2.0 that mask these regions to accurately detect pairwise relationships from IBD segment data. ERSA 2.0 also incorporates additional refinements to improve relationship detection accuracy for 1^st^- and 2^nd^-degree relationships. When error-prone IBD regions are masked, the relationship estimation methods in ERSA 2.0 perform well for a variety of IBD detection methods, including GERMLINE, fastIBD, and ISCA. Compared to high-density microarray data, WGS data provide a 5% to 15% increase in relationship detection power for 7^th^ through 12^th^-degree relationships. Whole-exome data perform substantially worse than high-density microarray data for this purpose. Our results demonstrate that ERSA 2.0 can detect relationships as distant as 12^th^ degree and has high power to detect relationships as distant as 8^th^ degree from whole-genome sequence data.

## Materials and Methods

### Whole-Genome Sequence Data

We included 258 individuals from 30 families and 46 unrelated individuals (34 Europeans, 4 Mexican-Americans, and 8 East Asians) in this study. We evaluated population structure for each unrelated individual and for one member of each family by performing principal components analysis (PCA) that incorporated HapMap population samples [22]. Of the 30 pedigrees, 25 clustered with Europeans, 3 with Mexican-Americans, and 2 with East Asians (Figure S2). The 30 pedigrees include 1490 documented pairwise relationships (see Table 1). One of these pedigrees was CEPH Pedigree #1463, which consists of genomes of a seventeen-member, three-generation pedigree, with publically available data (ftp://ftp2.completegenomics.com/Pedigree_1463/). Complete Genomics performed all whole-genome sequencing.

### Ethics Statement

With the exception of the publicly available CEPH Pedigree #1463, all other pedigree datasets are protected by human subjects protocols approved by the Western Institutional Review Board. Procedures followed were in accordance with institutional and national ethical standards of human experimentation. Proper informed consent was obtained. During subject recruitment, relationships were determined by interview and recorded.

### Pedigree Simulations

We simulated non-founder whole-genome data from fifteen-generation families (Figure S1), selecting founders randomly from the unrelated individuals of European ancestry described above. The whole genomes of two offspring were simulated in each generation. Genotypes of non-founders were obtained by simulating meiosis (recombination points were randomly selected based on the recombination rate map in [23]) and de-novo mutation with an expected rate of 1e-7. Sequencing errors were added to all non-founder genomes with an error rate of 0.001 per polymorphic site. There were 1035 pairs of individuals in each family, containing 330 unrelated pairs, 75 first-degree relationships (60 parent-offspring and fifteen full sibling pairs), 84 second-degree relationships, 78 third-degree relationships, 72 fourth-degree relationships, and 66 fifth-degree relationships.

### IBD Detection

We used ISCA to infer pairwise IBD1 and IBD2 segment estimates from unphased SNV data. We used Beagle and fastIBD to compute IBD estimates from unphased SNV data separately for each population. Each population combined European, Mexican-American, or East Asian control individuals with the pedigrees that clustered with those populations in PCA (Figure S2). We chose sequenced European genomes to serve as founders for each of the simulated pedigrees. The simulated pedigree genomes were phased with the European controls. Per the authors' recommendations, we ran fastIBD 10 times in each population and merged all segments within one megabase that overlapped between any of the 10 output files [13]; this additional step proved necessary for accurate relationship estimation in ERSA (Note that in our previous evaluation of fastIBD in ERSA we did not perform this step [10]). For GERMLINE, we first applied the grouping criteria above in three population analyses to phase each pedigree and each of the three control populations using Beagle [24], and then analyzed the phased data in GERMLINE.

We applied identical procedures for subsets of SNVs that lie within protein-coding exon boundaries or are Affymetrix 6.0 markers (Figure 3). For GERMLINE, we pruned the WGS datasets prior to phasing in Beagle. After generating IBD segments, we evaluated GERMLINE and fastIBD in ERSA 2.0. We estimated relationships for every pair of individuals within the pedigrees, using the appropriate control population identified in Figure S2.

### ERSA

ERSA models the distribution of IBD segments between two individuals in a maximum likelihood framework. The null model assumes that the size and number of IBD segments follow an empirical distribution approximated from the control population. Under the alternative model, some IBD segments may follow the control population distribution, but one or more segments follow a theoretical distribution derived according to a hypothesized recent relationship. Let *a* equal the number of shared ancestors and *d* equal the total number of meioses that separate the two individuals for the proposed relationship. For each pair of individuals, ERSA calculates the maximum likelihood for each possible relationship to identify the most likely relationship for that pair. We use the chi-square approximation to the maximum likelihood ratio to establish confidence intervals and to test for significance. This test has two degrees of freedom. One degree of freedom results from a parameter describing the number of segments that are attributable to hypothesized relationship for the pair of individuals (the remaining segments are attributed to the population distribution). A second degree of freedom results from the parameters *d* and *a*, which act approximately as a single parameter for most values of *d*. For direct ancestor-descendant relationships, a = 0. In ERSA 1.0, we assume that, for most relationships, the length *l* of an IBD segment inherited from the proposed relationship is exponentially distributed with mean equal to
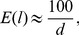
(1)in cM [25]. This approximation assumes that only recombination can break up an IBD segment. Because IBD segments are also broken at chromosomal boundaries,
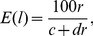
(2)where *c* is the number of autosomes and *r* is the expected number of recombination events per generation (r∼ = 35 in humans [23]). As d increases, Eq. 2 approaches Eq. 1, and thus Eq. 1 is a close approximation for distant relationships but is less accurate for close relationships. ERSA 2.0 uses Eq. 2 when *a* is equal to 1 or 0, resulting in an improvement in accuracy for closely related individuals (Figure S8). Empirically, we observed that Eq. 2 slightly reduced relationship estimation accuracy when *a* is equal to 2, perhaps due to minor biases in the estimated IBD lengths in GERMLINE and ISCA. Thus, we continue to use Eq. 1 for models where *a* is equal to 2. Both versions assume that the number of IBD segments, *n*, is Poisson distributed with mean equal to
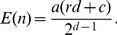
(3)


Modifications of these formulas for specific relationships are described below. Hill and White have very recently employed simulations to derive precise estimates for the joint distribution of the number and length of shared segments for a wide range of relationships [26]. These distributions are likely to provide further improvement in relationship detection accuracy in the future.

Although related individuals may sometimes share short IBD segments, such segments can be difficult to distinguish from more distant IBD segments that would be shared by unrelated members of the population. Thus, we typically set a minimum IBD segment length, *t*, and ignore all segments smaller than this length. By default, *t* is equal to 2.5. Whenever *t* is greater than 0, all formulas are adjusted to condition on the probability that IBD segment lengths are greater than or equal to *t* [10].

All ERSA results were with confidence level = 0.999 and *α* = 0.001. Reported power estimates were the percentage of related pairs that were correctly predicted to be related at *α* = 0.001.

### Genomic Region Masking

To mask genomic regions potentially prone to false-positive IBD, we first evaluate the distribution of IBD segments in a control population. Genomic regions are masked from the analysis if the ratio of observed to expected total IBD segment length exceeds a specified threshold, *h*. By default, *h* is equal to 4, but we observed similar results for values of *h* between 2 and 6 (Figure S9). The total IBD segment length equals the sum of all pairwise IBD segments that overlap a genomic region, with the segments truncated at the region boundaries. The expected total IBD segment length is calculated under the assumption that pairwise IBD segments in the population are distributed uniformly across the genome. Let *m* equal the summed length of all masked genomic regions, in cM. We subtract *m*/100 from *r* in all models to account for recombination events that cannot be observed. For each IBD segment, the length of the masked region is subtracted from the length of the IBD segment if the IBD segment wholly contains the region and extends at least *b* base pairs past the beginning and end of the region. By default, *b* is equal to 1 Mb. All other IBD segments that cross a masked region are truncated at the region boundary. Genomic region masking is an optional parameter in ERSA 2.0 (mask_common_shared_regions) that is inactive by default.

### Parent-Offspring Relationships (a = 0, d = 1)

Because parents and offspring are IBD1 throughout the entire genome, there is no stochasticity in the number and lengths of IBD segments. Therefore, for both versions of ERSA, parent-offspring is reported as the most likely relationship if the total IBD segment length is at least *z* standard deviations above the expected total segment length of a full-sibling relationship (0.75). By default, *z* is equal to 2.33.

### Other Direct Ancestor-Descendant Relationships (a = 0, d>1)

Other than parent-offspring, direct ancestor-descendant relationships (e.g. grandparent-grandchild) were not explicitly modeled in ERSA 1.0. The primary difference in IBD segment distribution between an ancestor-descendant relationship and a relationship with a shared ancestor is that recombination events in the first generation cannot be detected in a pairwise comparison unless complete phase information is available. ERSA 2.0 accounts for this difference with the following equations:
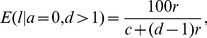
(4)and
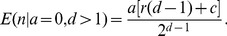
(5)


### Full-Sibling Relationships (a = 2, d = 2)

Because GERMLINE and fastIBD do not differentiate between IBD1 and IBD2, regions of IBD2 among full-siblings are merged with their flanking IBD1 segments and are reported as a single, larger IBD segment. Multiple IBD2 segments can be joined together in this manner. Let *k* equal the number IBD1 segments that have been bioinformatically merged. Conditioned on *k* and ignoring chromosomal boundaries, *l* follows a gamma distribution with shape parameter equal to *k*+1. ERSA 1.0 approximated the distribution of *l* using the maximum likelihood estimate of *k* with a single gamma distribution, which introduced an additional free parameter in the sibling model relative to other relationship models. To eliminate this free parameter, ERSA 2.0 assumes that *l* is distributed according to a mixture of gammas by summing over possible values of *k*. The likelihood of *l* is equal to:
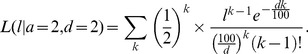
(6)


The expected number of IBD segments for full sibling relationships in both versions is:
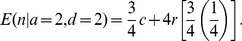
(7)


ERSA 2.0 now has the option of evaluating full-sibling models using IBD2 segment data. ERSA assumes that all overlapping IBD1 segments are merged into a single segment, and that each IBD2 segment is reported separately as an additional overlapping segment, which matches the output format that we generated from ISCA. Conditioned on the total length of IBD1 segments, *T*, the expected number of IBD2 segments under the null model is equal to the unconditional number of expected IBD1 segments multiplied by *T*/100*r*. The expected length of an IBD2 segment under the null model follows the empirical distribution for IBD1 segments. Under the alternative model, the number of IBD2 segments, *n_2_*, is approximately Poisson distributed with mean equal to
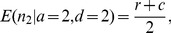
(8)and the expected length of an IBD2 segment is approximately exponentially distributed with mean equal to 25 cM. The IBD2 option (‘use_ibd2_siblings’) was used for all ISCA analyses.

### Avuncular Relationships (a = 2, d = 3)

All segments that are IBD2 between siblings must be IBD1 in an avuncular relationship involving one sibling and the offspring of the other sibling. For each such segment, an additional IBD1 segment in the siblings may be inherited by the offspring with probability of 0.5 if the following two events occur: 1) a recombination event occurs within the segment in the offspring (probability of approximately 0.5) and 2) an IBD1 segment does not flank a second IBD2 segment (probability of 0.5). All IBD1 segments in the siblings that are not part of an IBD2 segment are broken into two segments by recombination in the offspring with probability of approximately 0.5, in which case one of the segments are shared, and are otherwise are inherited in the offspring with probability 0.5. This leads to the following expression:
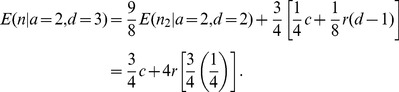
(9)


Thus, expected number of segments in an avuncular relationship is equal to the expected number in a full-sibling relationship (Eq. 7). This correction was implemented in ERSA 2.0 (ERSA 1.0 erroneously applied Eq 3 to avuncular models). Both versions approximate the distribution of IBD segment lengths in an avuncular relationship using Eq. 1 and assuming an exponential distribution.

### RELPAIR Analysis

We used RELPAIR to estimate relationships for all the pairs of individuals to which we applied ERSA 2.0. From the whole-genome data sets, we extracted 9999 well-spaced, relatively independent biallelic SNP loci with minor allele frequency >20%. Allele frequencies and linkage disequilibrium for all loci were assessed in the 34 unrelated CEU individuals using PLINK [27]. Linkage disequilibrium between loci was minimized by pruning correlated SNP loci (PLINK –indep, variance inflation factor up to 1.5 allowed, analysis conducted in windows of 400 SNPs, step size 10 SNPs.) Remaining closely-spaced SNP loci were removed until the target of 9999 SNPs was reached.

### Software

The relationship identification methods described above are implemented in the software package ERSA 2.0, which is freely available for academic use (www.hufflab.org). The software for ISCA is available at http://familygenomics.systemsbiology.net/software. The pedigree simulation programs are available in http://caballero.github.io/FakeFamily/.

## Supporting Information

Figure S1 A simulated 46-member, 15-generation pedigree. A square represents a male and a circle represents a female. Green symbols indicate founders that were sequenced by CGI, and purple symbols indicate children whose genotypes were simulated.
(PDF)

Figure S2 Principal component analysis (PCA) of individuals with whole-genome sequence data from this study. (A) Includes European controls, (B) includes East Asian controls, and (C) includes Mexican controls. Individuals labeled with “CG-” are from the Complete Genomics Diversity panel. “Unrelated-CEU(controls) are the 34 additional European individuals sequenced in this study. The three red circles indicate the three population groups used to match pedigrees and controls.
(PDF)

Figure S3 Performance of relationship estimation in 30 sequenced families without masking error prone IBD regions using (A) GERMLINE-ERSA2.0, (B) fastIBD-ERSA2.0, and (C) ISCA-ERSA2.0. Area of the circles indicates the percentage of individual pairs whose estimated degrees of relationship are exactly the same as real relationship. FS: full sibling. PO: parent offspring. UN: unrelated individuals.
(PDF)

Figure S4 Relationship estimation power in 30 sequenced families. Error bar indicates the 95% confidence level estimated from the binomial distribution. For GERMLINE and ISCA, ERSA 2.0 “not masked” power estimates are biased due to inflated Type I error rates resulting from spurious IBD.
(PDF)

Figure S5 Performance of relationship estimation in simulated WGS datasets (sequencing error rate = 0.001). (A) “GERMLINE-ERSA2.0” with masking background IBD. (B) “GERMLINE-ERSA2.0” without masking background IBD. (C) “fastIBD -ERSA2.0” with masking background IBD. (D) “fastIBD-ERSA2.0” without masking background IBD. (E) “ISCA-ERSA2.0” with masking background IBD. (F) “ISCA-ERSA2.0” without masking background IBD.
(PDF)

Figure S6 Power of relationship estimation in simulated WGS datasets (sequencing error rate = 0.001). GERMLINE, fastIBD and ISCA were respectively run on WGS markers to infer IBD segments, and then ERSA 2.0 was run to predict relationship degree. RELPAIR was run using 9999 well-spaced, relatively independent biallelic SNP loci (see Materials and Methods). For GERMLINE and ISCA, ERSA 2.0 “not masked” power estimates are biased due to inflated Type I error rates resulting from spurious IBD.
(PDF)

Figure S7 Regions where excess IBD is detected by 34 CEU or 20 new CEU control genomes. (A)–(C) GERMLINE, fastIBD, and ISCA results for 34 CEU genomes. (D)–(F) GERMLINE, fastIBD, and ISCA results for 20 new CEU genomes.
(PDF)

Figure S8 Comparison between ERSA 1.0 and ERSA 2.0. Exact prediction accuracy for (A) true pedigrees and (B) simulated pedigrees. Power for detecting related pairs in (C) true pedigrees and (D) simulated pedigrees.
(PDF)

Figure S9 Proportion of detected relationships among unrelated controls at different masking cutoffs. The dash line indicates ERSA 2.0's default cutoff (4). Results without masking are show on the right.
(PDF)

Figure S10 Comparison of ERSA 2.0 's performance for real and simulated pedigrees. (A) Exact prediction accuracy. (B) Power. They only show consistent relationship degree (1^st^–6^th^ and 9^th^–11^th^) in both datasets.
(PDF)

Table S1 Prediction accuracy and power of ERSA 2.0 with masking on real families. Numerical values in the table are results for WGS data, numerical values in parentheses are results for “SNP microarray” and “exon” data. Three parameter setting of BEAGLE 4 preview version [28] were used: A) ibdlength = 0.5, IBD segments are not merged; B) ibdlength = 0.5, run program 10 times and merge all segments within two Mb; C) ibdwindow = 304, ibdtrim = 228, overlap = 2281, ibdlength = 0.5, run program 10 times and merge all segments within two Mb.
(DOCX)

Table S2 Consistency in spurious IBD regions detected by each method. We only considered spurious regions whose observed/expected ratio is larger than 4 and genetic longer is longer than 1 MB. (Numerical values in parentheses are results for comparing regions that are longer than 2 MB). Pairwise correlation between two methods was calculated by “Jaccard similarity coefficient”, the ratio of overlapped region length to total region length. CEU (A) refers to the 34 European control genomes described in the main text. CEU (B) is an additional sample of 20 unrelated Europeans.
(DOCX)

Table S3 Excess IBD regions in Table 3 and their observed/expected ratio in the 34 European control genomes described in the main text (A) and 20 additional unrelated Europeans (B).
(DOCX)

Table S4 Comparison regions identified in Table 3 with regions influenced by positive selection [16].
(DOCX)

Table S5 Comparison regions identified in Table 3 with long-range haplotypes reported by Gusev et. al. [17].
(DOCX)

Table S6 Comparison regions identified in Table 3 with long-range haplotypes reported by Price et. al. [18].
(DOCX)

## References

[1] Boehnke M, Cox NJ (1997) Accurate inference of relationships in sib-pair linkage studies. Am J Hum Genet 61: 423–429. 9311748 10.1086/514862 PMC1715905

[2] Voight BF, Pritchard JK (2005) Confounding from cryptic relatedness in case-control association studies. PLoS Genet 1: e32. 16151517 10.1371/journal.pgen.0010032 PMC1200427

[3] Kong A, Thorleifsson G, Gudbjartsson DF, Masson G, Sigurdsson A, et al (2010) Fine-scale recombination rate differences between sexes, populations and individuals. Nature 467: 1099–1103. 20981099 10.1038/nature09525

[4] Xing J, Watkins WS, Shlien A, Walker E, Huff CD, et al (2010) Toward a more uniform sampling of human genetic diversity: A survey of worldwide populations by high-density genotyping. Genomics 96: 199–210. 20643205 10.1016/j.ygeno.2010.07.004 PMC2945611

[5] Lin TH, Myers EW, Xing EP (2006) Interpreting anonymous DNA samples from mass disasters–probabilistic forensic inference using genetic markers. Bioinformatics 22: e298–306. 16873485 10.1093/bioinformatics/btl200

[6] Alvarez-Cubero MJ, Saiz M, Martinez-Gonzalez LJ, Alvarez JC, Eisenberg AJ, et al (2012) Genetic identification of missing persons: DNA analysis of human remains and compromised samples. Pathobiology 79: 228–238. 22722562 10.1159/000334982

[7] Thompson EA (1975) The estimation of pairwise relationships. Ann Hum Genet 39: 173–188. 1052764 10.1111/j.1469-1809.1975.tb00120.x

[8] Ehm MG WM (1996) Test statistic to detect errors in sib-pair relationships. Am J Hum Genet Suppl 69: A217. 10.1086/301668 PMC1376795 9443861

[9] Epstein MP, Duren WL, Boehnke M (2000) Improved inference of relationship for pairs of individuals. Am J Hum Genet 67: 1219–1231. 11032786 10.1016/s0002-9297(07)62952-8 PMC1288564

[10] Huff CD, Witherspoon DJ, Simonson TS, Xing J, Watkins WS, et al (2011) Maximum-likelihood estimation of recent shared ancestry (ERSA). Genome Res 21: 768–774. 21324875 10.1101/gr.115972.110 PMC3083094

[11] Henn BM, Hon L, Macpherson JM, Eriksson N, Saxonov S, et al (2012) Cryptic distant relatives are common in both isolated and cosmopolitan genetic samples. PLoS One 7: e34267. 22509285 10.1371/journal.pone.0034267 PMC3317976

[12] Gusev A, Lowe JK, Stoffel M, Daly MJ, Altshuler D, et al (2009) Whole population, genome-wide mapping of hidden relatedness. Genome Res 19: 318–326. 18971310 10.1101/gr.081398.108 PMC2652213

[13] Browning BL, Browning SR (2011) A fast, powerful method for detecting identity by descent. Am J Hum Genet 88: 173–182. 21310274 10.1016/j.ajhg.2011.01.010 PMC3035716

[14] Roach JC, Glusman G, Smit AF, Huff CD, Hubley R, et al (2010) Analysis of Genetic Inheritance in a Family Quartet by Whole-Genome Sequencing. Science 328: 636–9. 20220176 10.1126/science.1186802 PMC3037280

[15] Smit AFA, Hubley R. (2008–2010) RepeatModeler Open-1.0.

[16] Albrechtsen A, Moltke I, Nielsen R (2010) Natural selection and the distribution of identity-by-descent in the human genome. Genetics 186: 295–308. 20592267 10.1534/genetics.110.113977 PMC2940294

[17] Gusev A, Palamara PF, Aponte G, Zhuang Z, Darvasi A, et al (2012) The architecture of long-range haplotypes shared within and across populations. Mol Biol Evol 29: 473–486. 21984068 10.1093/molbev/msr133 PMC3350316

[18] Price AL, Weale ME, Patterson N, Myers SR, Need AC, et al (2008) Long-range LD can confound genome scans in admixed populations. Am J Hum Genet 83: 132–135 author reply 135–139. 18606306 10.1016/j.ajhg.2008.06.005 PMC2443852

[19] Tian C, Plenge RM, Ransom M, Lee A, Villoslada P, et al (2008) Analysis and application of European genetic substructure using 300 K SNP information. PLoS Genet 4: e4. 18208329 10.1371/journal.pgen.0040004 PMC2211544

[20] Roach JC, Glusman G, Smit AF, Huff CD, Hubley R, et al (2010) Analysis of genetic inheritance in a family quartet by whole-genome sequencing. Science 328: 636–639. 20220176 10.1126/science.1186802 PMC3037280

[21] Roach JC, Glusman G, Hubley R, Montsaroff SZ, Holloway AK, et al (2011) Chromosomal haplotypes by genetic phasing of human families. Am J Hum Genet 89: 382–397. 21855840 10.1016/j.ajhg.2011.07.023 PMC3169815

[22] Consortium TIH (2007) A second generation human haplotype map of over 3.1 million SNPs. Nature 449: 851–861. 17943122 10.1038/nature06258 PMC2689609

[23] McVean GA, Myers SR, Hunt S, Deloukas P, Bentley DR, et al (2004) The fine-scale structure of recombination rate variation in the human genome. Science 304: 581–584. 15105499 10.1126/science.1092500

[24] Browning SR, Browning BL (2007) Rapid and accurate haplotype phasing and missing-data inference for whole-genome association studies by use of localized haplotype clustering. Am J Hum Genet 81: 1084–1097. 17924348 10.1086/521987 PMC2265661

[25] Thomas A, Skolnick MH, Lewis CM (1994) Genomic mismatch scanning in pedigrees. IMA J Math Appl Med Biol 11: 1–16. 8057038 10.1093/imammb/11.1.1

[26] Hill WG, White IM (2013) Identification of pedigree relationship from genome sharing. G3 (Bethesda) 3: 1553–1571. 23893739 10.1534/g3.113.007500 PMC3755916

[27] Purcell S, Neale B, Todd-Brown K, Thomas L, Ferreira MA, et al (2007) PLINK: a tool set for whole-genome association and population-based linkage analyses. Am J Hum Genet 81: 559–575. 17701901 10.1086/519795 PMC1950838

[28] Browning BL, Browning SR (2013) Improving the Accuracy and Efficiency of Identity-By-Descent Detection in Population Data. Genetics 194 2: 459–471. 23535385 10.1534/genetics.113.150029 PMC3664855

