# Supplementary figures and images for: Relationship Estimation from Whole-Genome Sequence Data
Source: PLoS Genet. 2014 Jan 30;10(1):e1004144. doi: 10.1371/journal.pgen.1004144 (PMC3907355; doi:10.1371/journal.pgen.1004144)

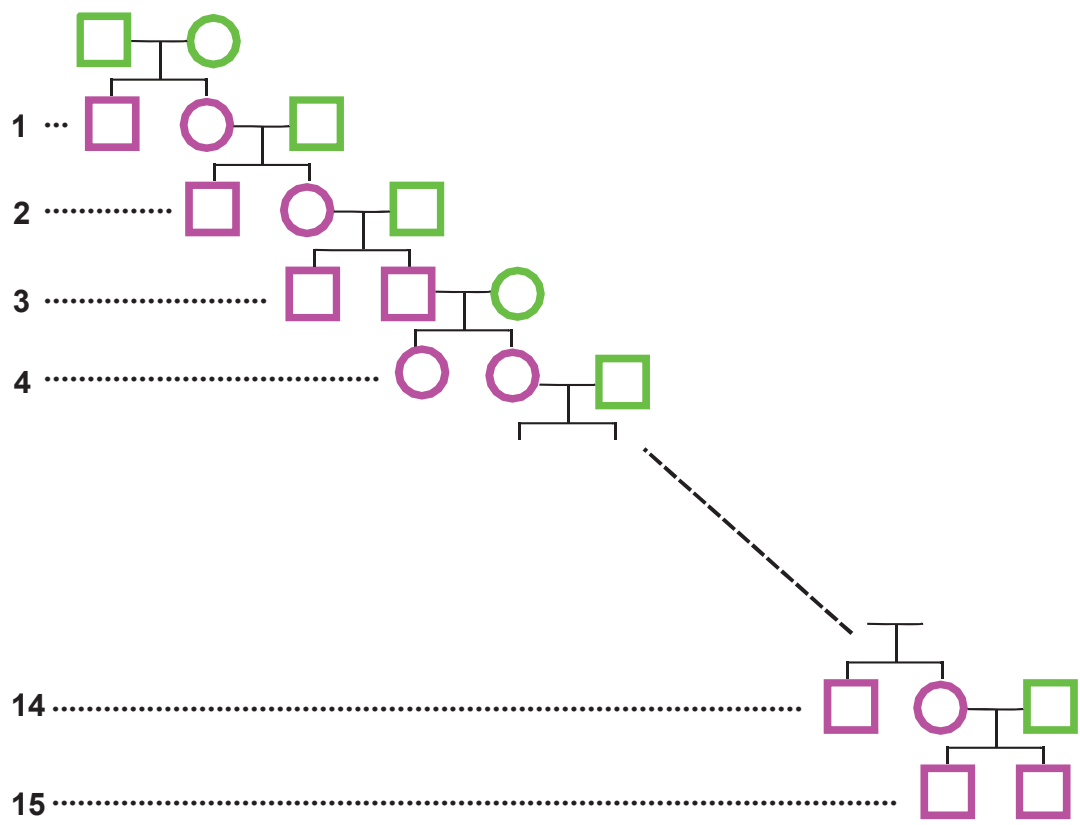

Supplement: Figure S1 — A simulated 46-member, 15-generation pedigree. A square represents a male and a circle represents a female. Green symbols indicate founders that were sequenced by CGI, and purple symbols indicate children whose genotypes were simulated. (PDF) [file pgen.1004144.s001.pdf]

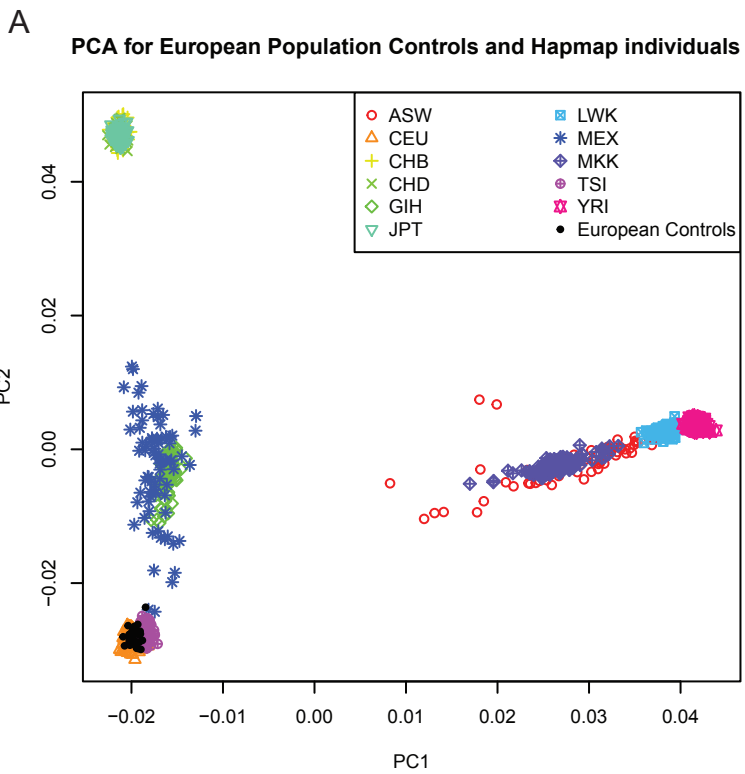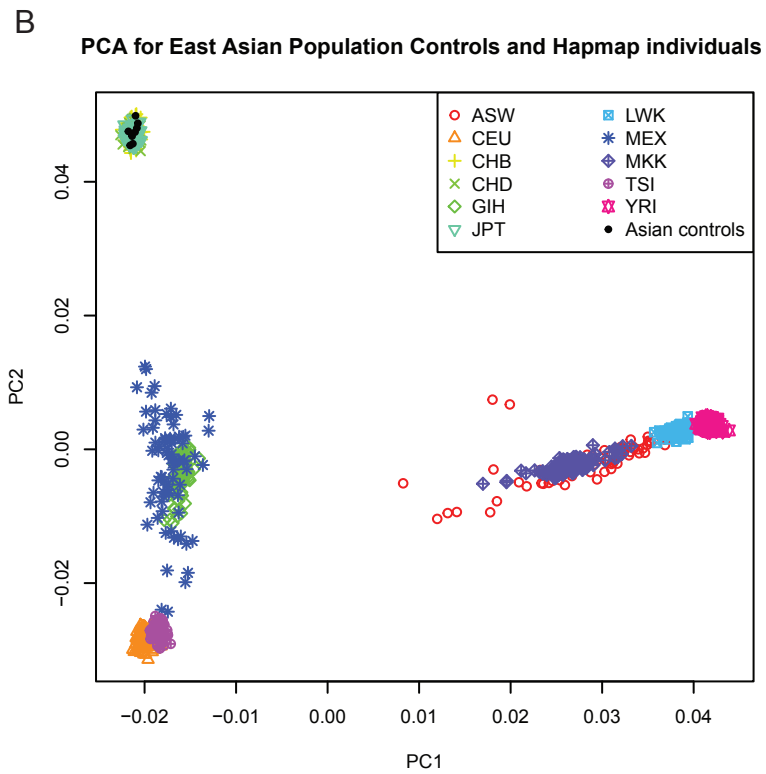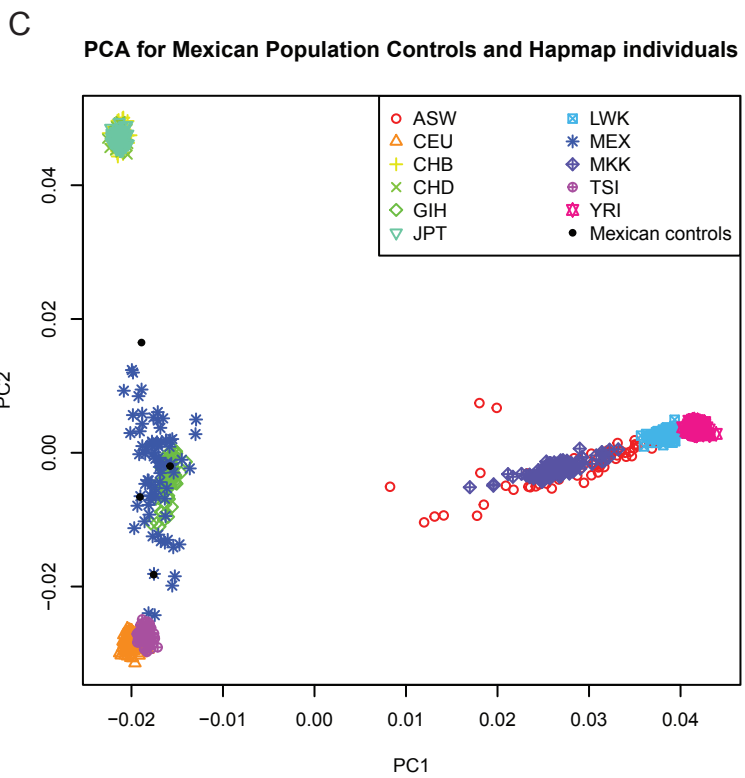

Supplement: Figure S2 — Principal component analysis (PCA) of individuals with whole-genome sequence data from this study. (A) Includes European controls, (B) includes East Asian controls, and (C) includes Mexican controls. Individuals labeled with “CG-” are from the Complete Genomics Diversity panel. “Unrelated-CEU(controls) are the 34 additional European individuals sequenced in this study. The three red circles indicate the three population groups used to match pedigrees and controls. (PDF) [file pgen.1004144.s002.pdf]

**A** GERMLINE+ERSA2.0 (not masked)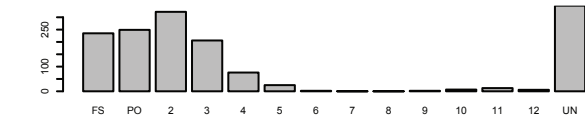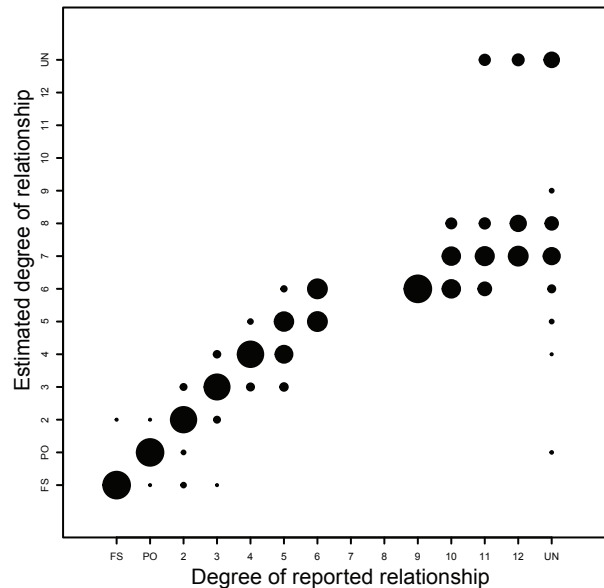**B** fastIBD+ERSA2.0 (not masked)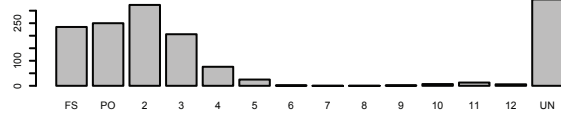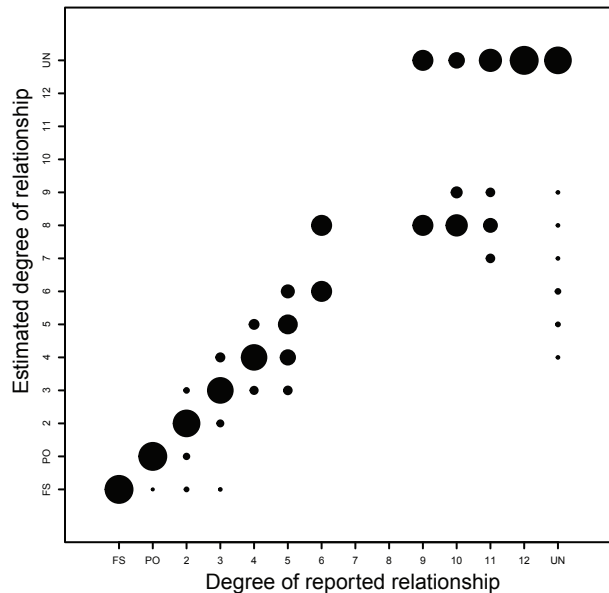**C** ISCA+ERSA2.0 (not masked)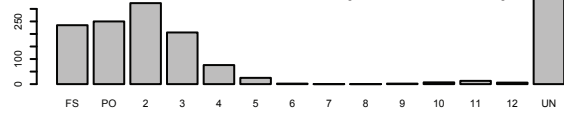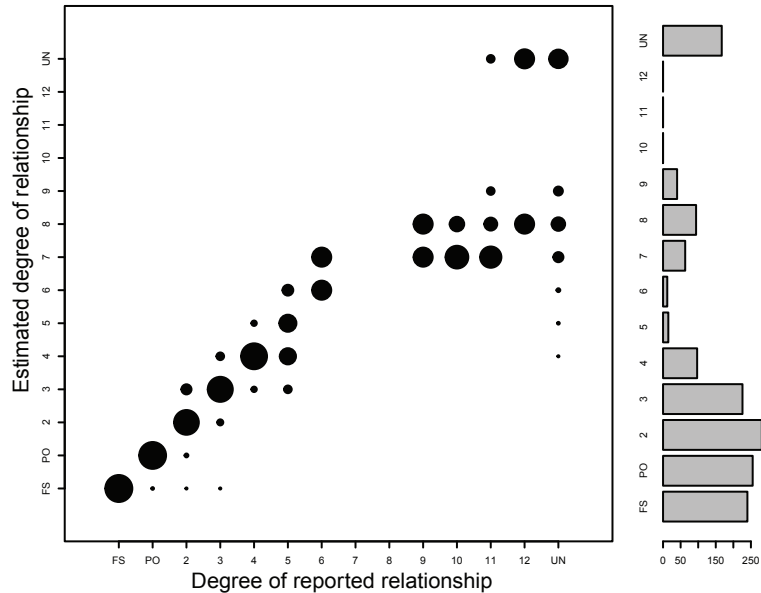

Supplement: Figure S3 — Performance of relationship estimation in 30 sequenced families without masking error prone IBD regions using (A) GERMLINE-ERSA2.0, (B) fastIBD-ERSA2.0, and (C) ISCA-ERSA2.0. Area of the circles indicates the percentage of individual pairs whose estimated degrees of relationship are exactly the same as real relationship. FS: full sibling. PO: parent offspring. UN: unrelated individuals. (PDF) [file pgen.1004144.s003.pdf]

**masked**

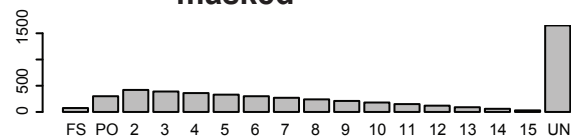

**A**

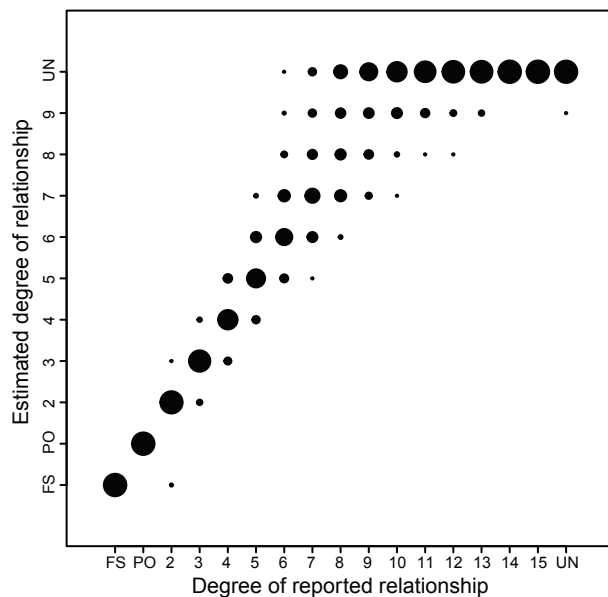

**GERMLINE**

**not masked**

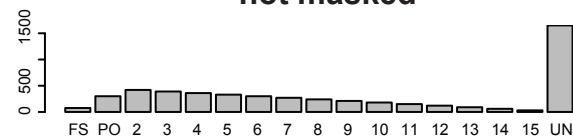

**B**

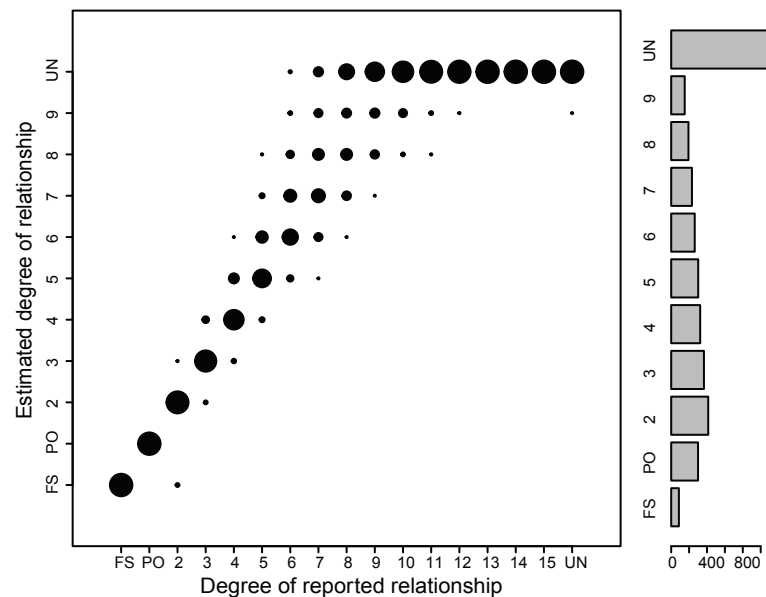

**C**

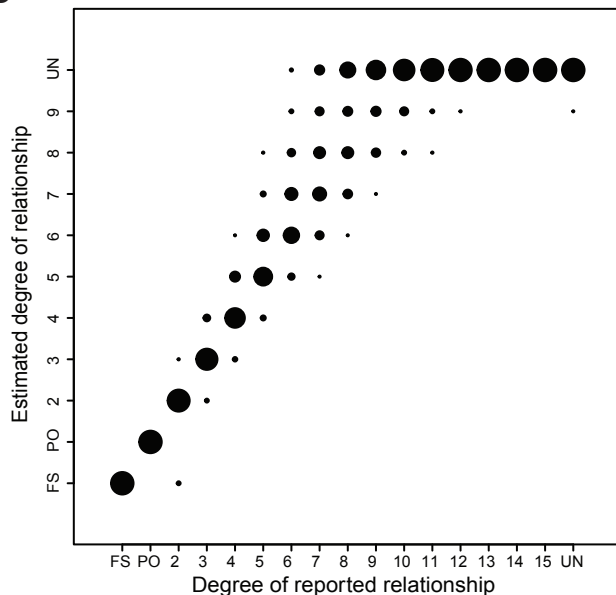

**fastIBD**

**D**

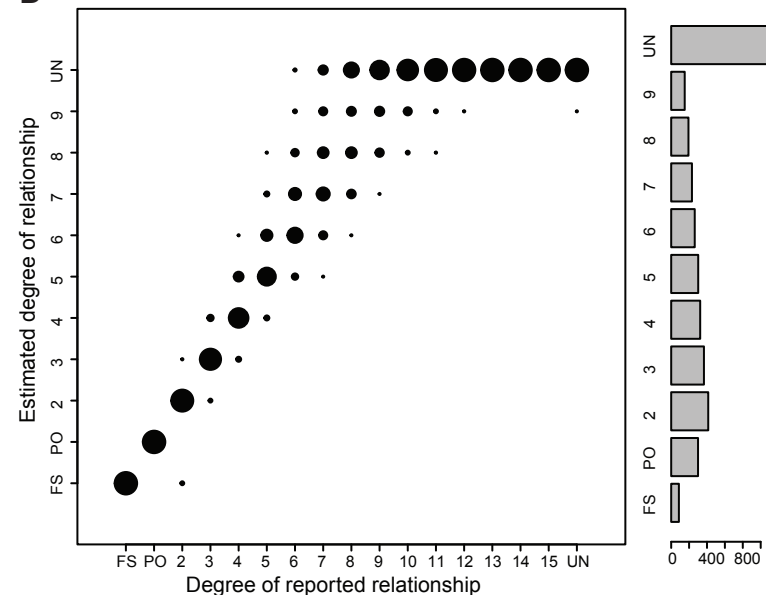

**E**

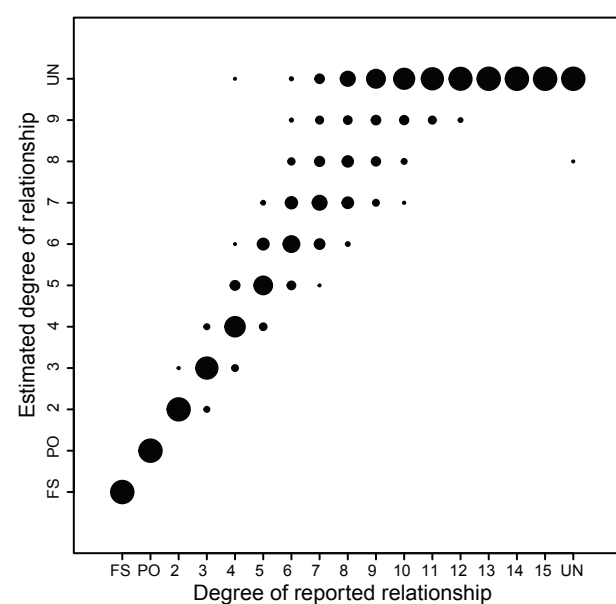

**ISCA**

**F**

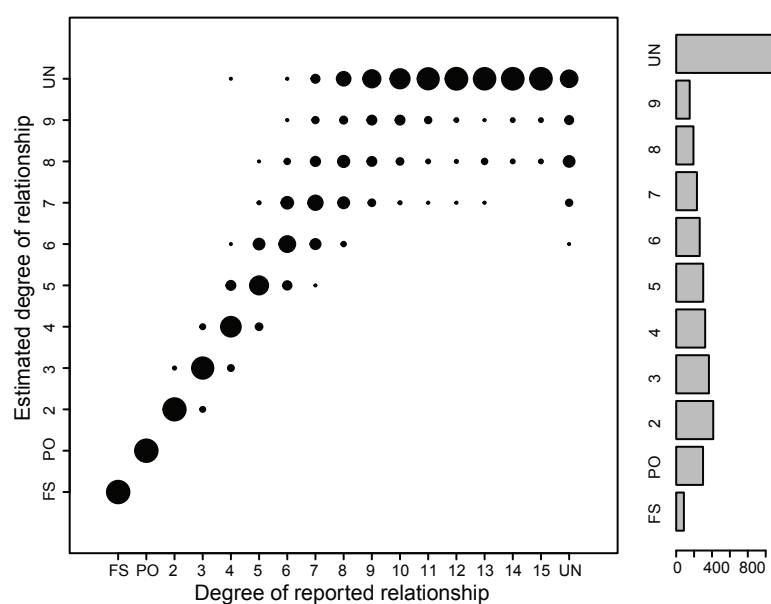

Supplement: Figure S5 — Performance of relationship estimation in simulated WGS datasets (sequencing error rate = 0.001). (A) “GERMLINE-ERSA2.0” with masking background IBD. (B) “GERMLINE-ERSA2.0” without masking background IBD. (C) “fastIBD -ERSA2.0” with masking background IBD. (D) “fastIBD-ERSA2.0” without masking background IBD. (E) “ISCA-ERSA2.0” with masking background IBD. (F) “ISCA-ERSA2.0” without masking background IBD. (PDF) [file pgen.1004144.s005.pdf]

power to detect a relationship

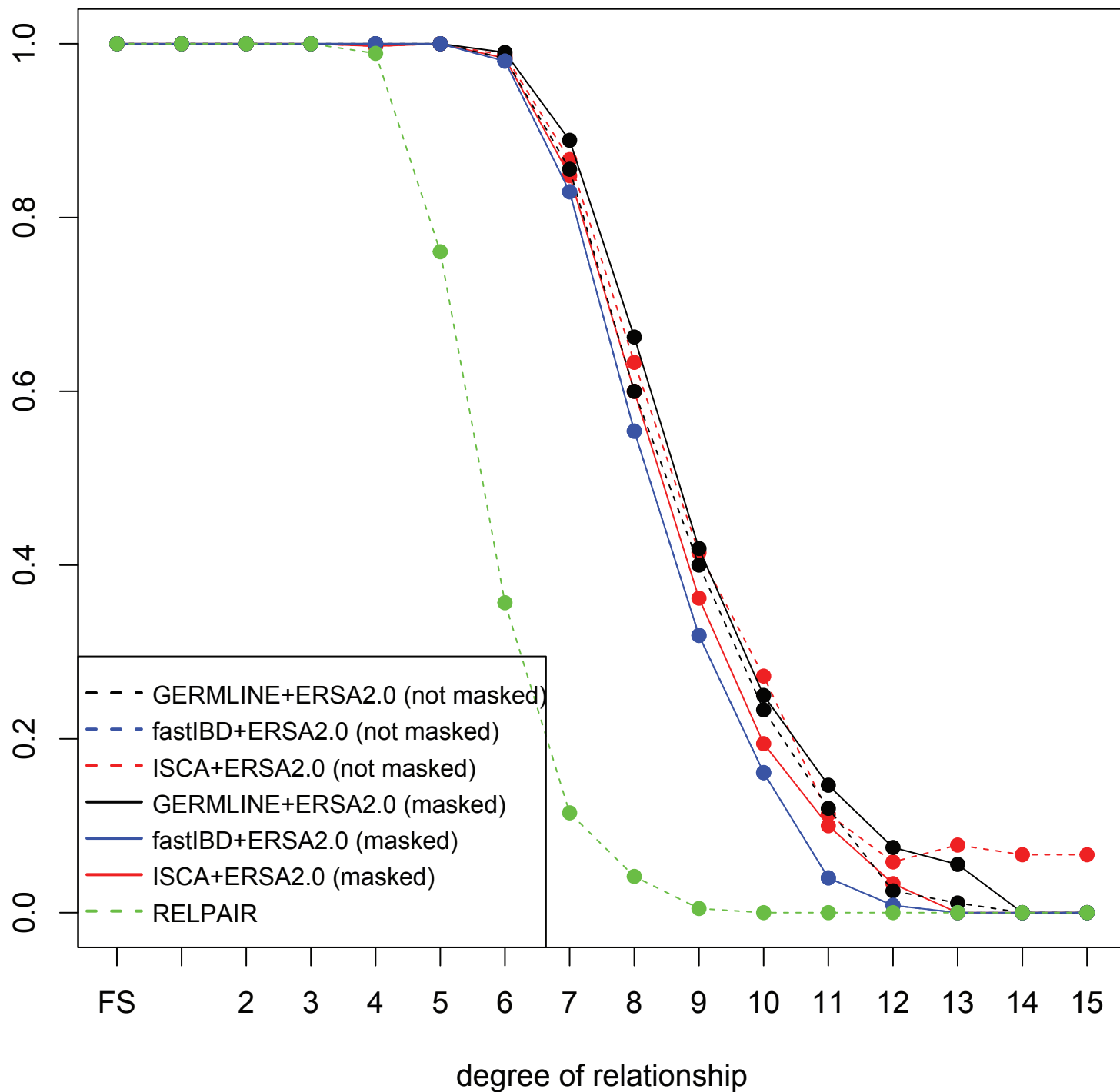

Supplement: Figure S6 — Power of relationship estimation in simulated WGS datasets (sequencing error rate = 0.001). GERMLINE, fastIBD and ISCA were respectively run on WGS markers to infer IBD segments, and then ERSA 2.0 was run to predict relationship degree. RELPAIR was run using 9999 well-spaced, relatively independent biallelic SNP loci (see Materials and Methods). For GERMLINE and ISCA, ERSA 2.0 “not masked” power estimates are biased due to inflated Type I error rates resulting from spurious IBD. (PDF) [file pgen.1004144.s006.pdf]

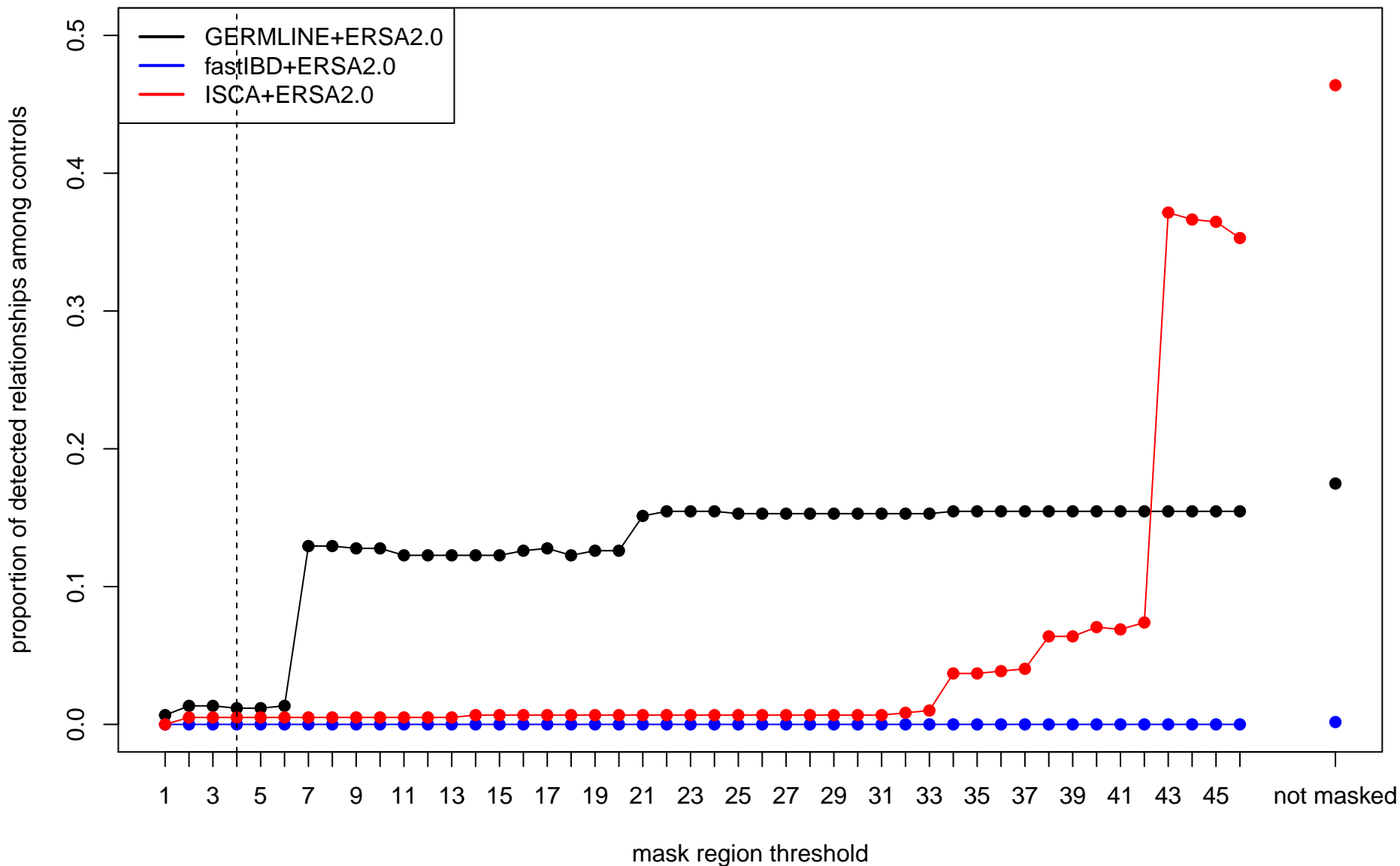

Supplement: Figure S9 — Proportion of detected relationships among unrelated controls at different masking cutoffs. The dash line indicates ERSA 2.0's default cutoff (4). Results without masking are show on the right. (PDF) [file pgen.1004144.s009.pdf]

**A****Exact prediction accuracy**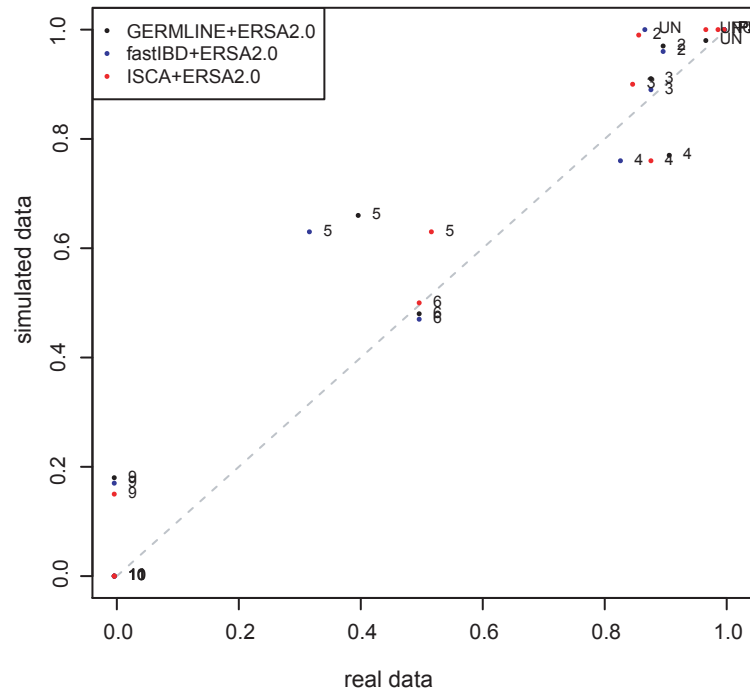**B****Power**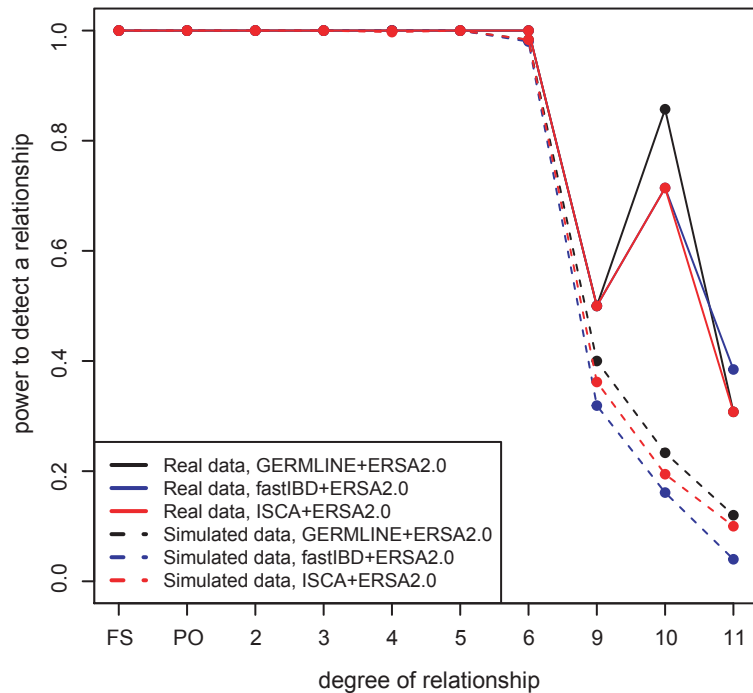

Supplement: Figure S10 — Comparison of ERSA 2.0 's performance for real and simulated pedigrees. (A) Exact prediction accuracy. (B) Power. They only show consistent relationship degree (1st–6th and 9th–11th) in both datasets. (PDF) [file pgen.1004144.s010.pdf]
