# Supplementary material for: Relationship Estimation from Whole-Genome Sequence Data
Source: PLoS Genet. 2014 Jan 30;10(1):e1004144. doi: 10.1371/journal.pgen.1004144 (PMC3907355; doi:10.1371/journal.pgen.1004144)
Supplement: Figure S4 — Relationship estimation power in 30 sequenced families. Error bar indicates the 95% confidence level estimated from the binomial distribution. For GERMLINE and ISCA, ERSA 2.0 “not masked” power estimates are biased due to inflated Type I error rates resulting from spurious IBD. (PDF) [file pgen.1004144.s004.pdf]

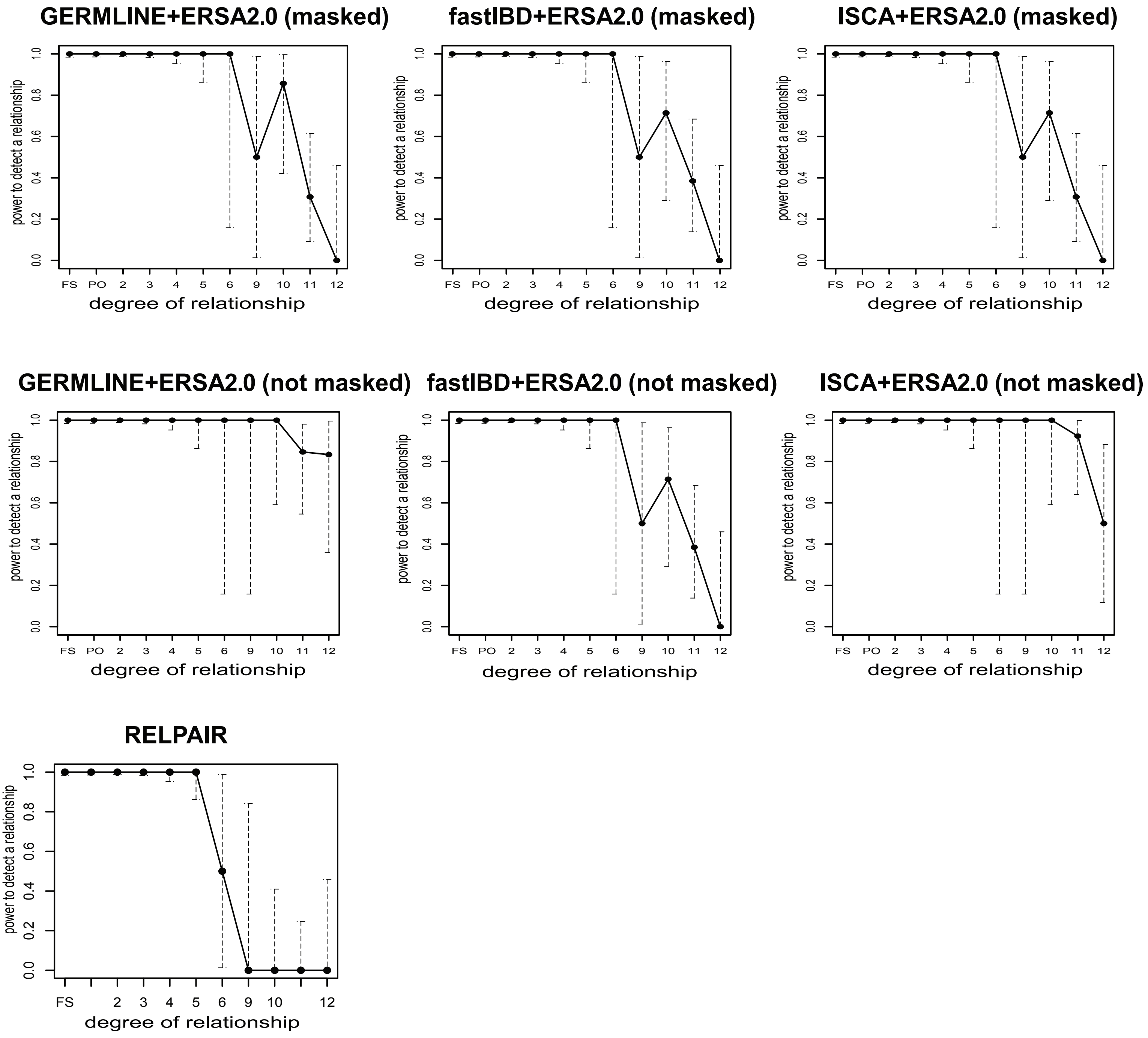
GERMLINE+ERSA2.0 (masked)
fastIBD+ERSA2.0 (masked)
ISCA+ERSA2.0 (masked)
GERMLINE+ERSA2.0 (not masked)
fastIBD+ERSA2.0 (not masked)
ISCA+ERSA2.0 (not masked)
RELPAIR
power to detect a relationship
degree of relationship
FS
PO
2
3
4
5
6
9
10
11
12
0.0
0.2
0.4
0.6
0.8
1.0
