# Supplementary material for: Relationship Estimation from Whole-Genome Sequence Data
Source: PLoS Genet. 2014 Jan 30;10(1):e1004144. doi: 10.1371/journal.pgen.1004144 (PMC3907355; doi:10.1371/journal.pgen.1004144)
Supplement: Figure S7 — Regions where excess IBD is detected by 34 CEU or 20 new CEU control genomes. (A)–(C) GERMLINE, fastIBD, and ISCA results for 34 CEU genomes. (D)–(F) GERMLINE, fastIBD, and ISCA results for 20 new CEU genomes. (PDF) [file pgen.1004144.s007.pdf]

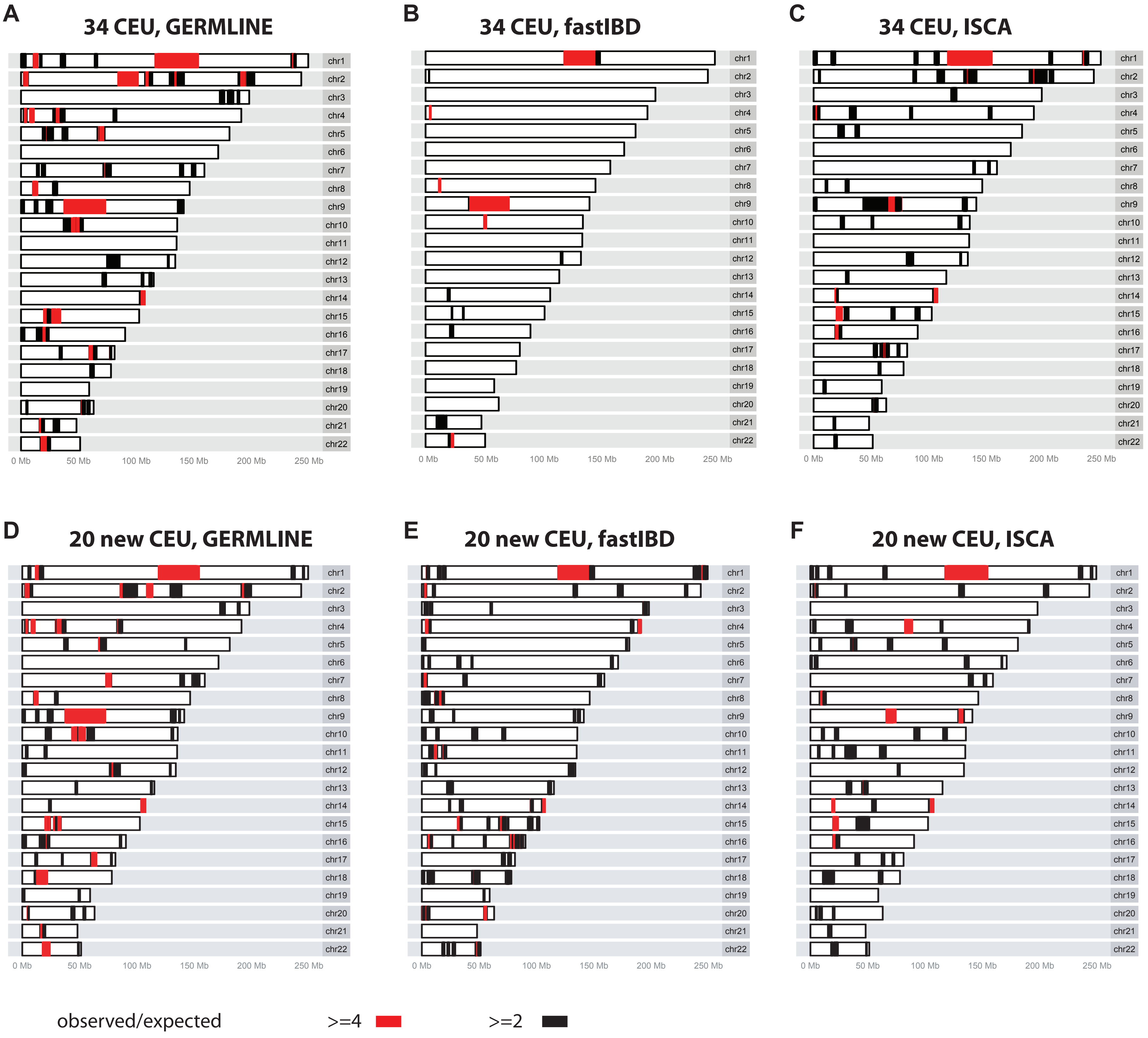
A
34 CEU, GERMLINE
B
34 CEU, fastIBD
C
34 CEU, ISCA
D
20 new CEU, GERMLINE
E
20 new CEU, fastIBD
F
20 new CEU, ISCA
chr1
chr2
chr3
chr4
chr5
chr6
chr7
chr8
chr9
chr10
chr11
chr12
chr13
chr14
chr15
chr16
chr17
chr18
chr19
chr20
chr21
chr22
0 Mb
50 Mb
100 Mb
150 Mb
200 Mb
250 Mb
observed/expected
>=4
>=2
