# Supplementary material for: Relationship Estimation from Whole-Genome Sequence Data
Source: PLoS Genet. 2014 Jan 30;10(1):e1004144. doi: 10.1371/journal.pgen.1004144 (PMC3907355; doi:10.1371/journal.pgen.1004144)
Supplement: Figure S8 — Comparison between ERSA 1.0 and ERSA 2.0. Exact prediction accuracy for (A) true pedigrees and (B) simulated pedigrees. Power for detecting related pairs in (C) true pedigrees and (D) simulated pedigrees. (PDF) [file pgen.1004144.s008.pdf]

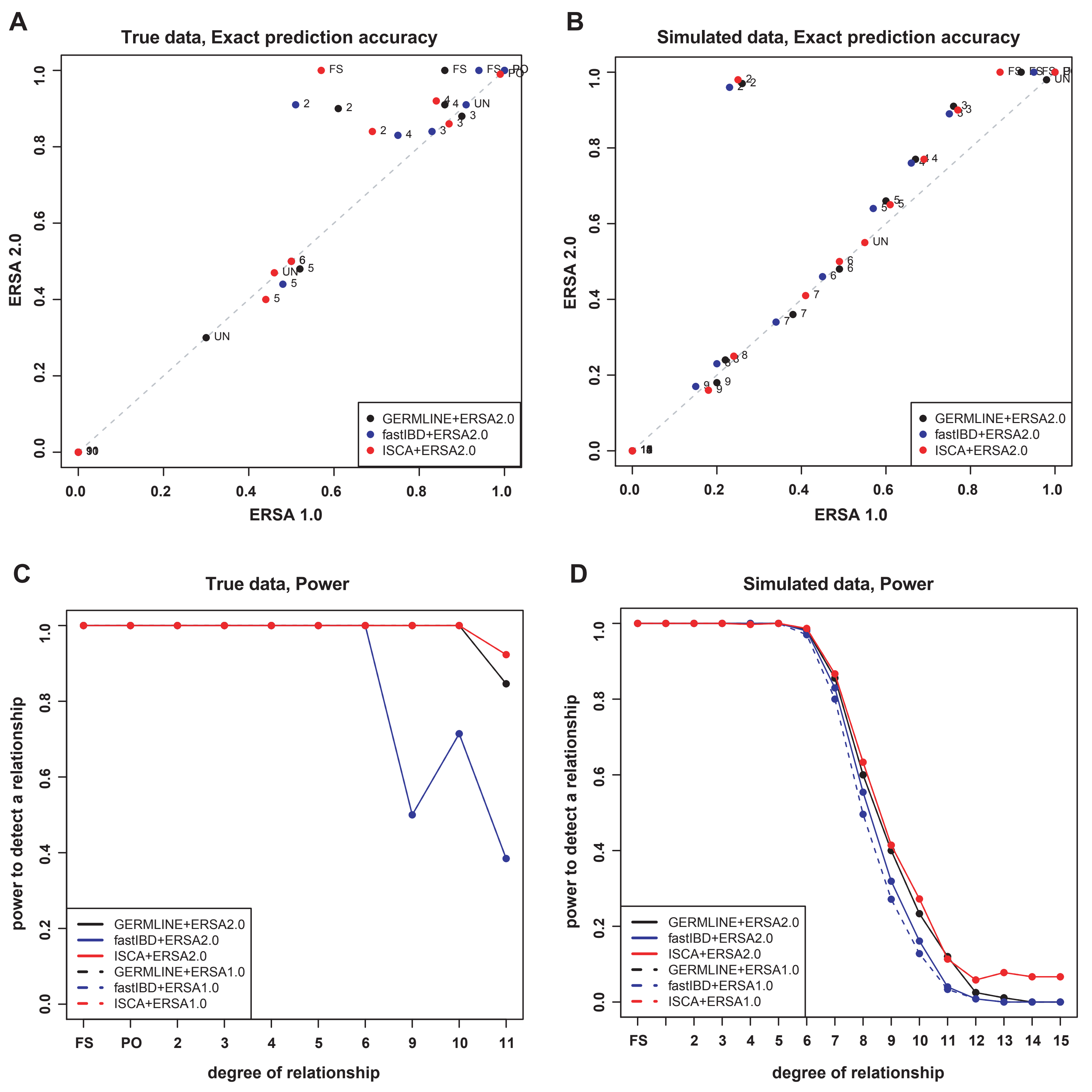
A
True data, Exact prediction accuracy
ERSA 2.0
ERSA 1.0
GERMLINE+ERSA2.0
fastIBD+ERSA2.0
ISCA+ERSA2.0
B
Simulated data, Exact prediction accuracy
ERSA 2.0
ERSA 1.0
GERMLINE+ERSA2.0
fastIBD+ERSA2.0
ISCA+ERSA2.0
C
True data, Power
power to detect a relationship
degree of relationship
GERMLINE+ERSA2.0
fastIBD+ERSA2.0
ISCA+ERSA2.0
GERMLINE+ERSA1.0
fastIBD+ERSA1.0
ISCA+ERSA1.0
D
Simulated data, Power
power to detect a relationship
degree of relationship
GERMLINE+ERSA2.0
fastIBD+ERSA2.0
ISCA+ERSA2.0
GERMLINE+ERSA1.0
fastIBD+ERSA1.0
ISCA+ERSA1.0
