# Supplementary material for: Relationship Estimation from Whole-Genome Sequence Data
Source: PLoS Genet. 2014 Jan 30;10(1):e1004144. doi: 10.1371/journal.pgen.1004144 (PMC3907355; doi:10.1371/journal.pgen.1004144)
Supplement: Table S1 — Prediction accuracy and power of ERSA 2.0 with masking on real families. Numerical values in the table are results for WGS data, numerical values in parentheses are results for “SNP microarray” and “exon” data. Three parameter setting of BEAGLE 4 preview version [28] were used: A) ibdlength = 0.5, IBD segments are not merged; B) ibdlength = 0.5, run program 10 times and merge all segments within two Mb; C) ibdwindow = 304, ibdtrim = 228, overlap = 2281, ibdlength = 0.5, run program 10 times and merge all segments within two Mb. (DOCX) [file pgen.1004144.s011.docx]

|  | **FS** | **PO** | **2^nd^-**  **degree** | **3^rd^-**  **degree** | **4^th^-**  **degree** | **5^th^-**  **degree** | **6^th^-**  **degree** | **9^th^-**  **degree** | **10^th^-**  **degree** | **11^th^-degree** | **12 ^th^-degree** | **Unrelated** |
| --- | --- | --- | --- | --- | --- | --- | --- | --- | --- | --- | --- | --- |
| **BEAGLE 4 (A) accuracy** | 0.95 (0.65,  0.01) | 0.81 (0.18,  0.00) | 0.12  (0.22,  0.00) | 0.98  (0.73,  0.04) | 0.75  (0.53,  0.00) | 0.32 (0.04,  0.04) | 0.00  (0.50,  0.00) | 0.00  (0.00,  0.00) | 0.00  (0.00,  0.00) | 0.00  (0.00,  0.00) | 0.00  (0.00,  0.00) | 0.97  (0.97,  0.99) |
| **BEAGLE 4 (B) accuracy** | 1.00 (0.85, 0.02) | 0.99 (0.34, 0.00) | 0.89 (0.66, 0.00) | 0.85 (0.60, 0.04) | 0.79 (0.28, 0.00) | 0.48 (0.04, 0.04) | 0.00  (0.50,  0.00) | 0.00  (0.00,  0.00) | 0.00  (0.00,  0.00) | 0.00  (0.00,  0.00) | 0.00  (0.00,  0.00) | 0.95  (0.97,  0.99) |
| **BEAGLE 4 (C) accuracy** | 0.99 (0.35,  0.00) | 0.93  (0.09,  0.00) | 0.83 (0.28,  0.00) | 0.85  (0.26,  0.00) | 0.75 (0.08,  0.00) | 0.36  (0.00,  0.00) | 0.50  (0.00,  0.00) | 0.00  (0.00,  0.00) | 0.00  (0.00,  0.00) | 0.00  (0.00,  0.00) | 0.00  (0.00,  0.00) | 0.96  (0.98,  1.00) |
| **GERMLINE accuracy** | 1.00  (1.00,  0.96) | 1.00  (1.00,  0.94) | 0.90  (0.90,  0.80) | 0.88  (0.86,  0.72) | 0.91  (0.89,  0.74) | 0.40  (0.48,  0.40) | 0.50  (0.50,  0.00) | 0.00  (0.00,  0.00) | 0.00  (0.00,  0.00) | 0.00  (0.00,  0.00) | 0.00  (0.00,  0.00) | 0.97  (0.97,  0.96) |
| **fastIBD**  **accuracy** | 1.00  (1.00,  1.00) | 0.99  (0.99,  0.99) | 0.73  (0.73,  0.73) | 0.90  (0.90,  0.90) | 0.78  (0.78,  0.78) | 0.48  (0.48,  0.48) | 0.50  (0.50,  0.50) | 0.00  (0.00,  0.00) | 0.00  (0.00,  0.00) | 0.00  (0.00,  0.00) | 0.00  (0.00,  0.00) | 0.94  (0.94,  0.94) |
| **ISCA**  **accuracy** | 1.00  (1.00,  1.00) | 0.99  (0.99,  0.99) | 0.86  (0.93,  0.80) | 0.85  (0.83,  0.21) | 0.88  (0.79,  0.05) | 0.52  (0.44,  0.08) | 0.50  (0.50,  0.00) | 0.00  (0.00,  0.00) | 0.00  (0.00,  0.00) | 0.00  (0.00,  0.00) | 0.00  (0.00,  0.00) | 0.97  (0.98,  0.99) |
| **BEAGLE 4 (A) power** | 1.00  (1.00,  1.00) | 1.00  (1.00,  1.00) | 1.00  (1.00,  0.96) | 1.00  (1.00,  0.83) | 1.00  (1.00,  0.34) | 0.92  (0.76,  0.16) | 1.00  (0.50,  0.00) | 0.50  (0.50,  0.00) | 0.86  (0.71,  0.00) | 0.46  (0.30, 0.00) | 0.00  (0.00,  0.00) |  |
| **BEAGLE 4 (B) power** | 1.00  (1.00,  1.00) | 1.00  (1.00,  1.00) | 1.00  (1.00,  0.96) | 1.00  (1.00,  0.83) | 1.00  (1.00,  0.34) | 1.00  (0.76,  0.16) | 1.00  (1.00,  0.00) | 0.50  (0.50,  0.00) | 0.57  (0.71,  0.00) | 0.15  (0.31,  0.00) | 0.00  (0.00,  0.00) |  |
| **BEAGLE 4 (C) power** | 1.00  (1.00,  0.98) | 1.00  (0.85,  0.90) | 1.00  (0.90,  0.82) | 1.00  (0.83,  0.68) | 1.00  (0.76,  0.18) | 1.00  (0.48,  0.08) | 1.00  (0.00,  0.00) | 0.50  (0.50,  0.00) | 0.43  (0.57,  0.00) | 0.08  (0.23,  0.00) | 0.00  (0.00,  0.00) |  |
| **GERMLINE power** | 1.00  (1.00,  1.00) | 1.00  (1.00,  1.00) | 1.00  (1.00,  1.00) | 1.00  (1.00,  1.00) | 1.00  (1.00,  1.00) | 1.00  (1.00,  1.00) | 1.00  (1.00,  0.00) | 0.50  (0.50,  0.50) | 0.86  (0.57,  0.00) | 0.31  (0.31,  0.15) | 0.00  (0.00,  0.00) |  |
| **fastIBD**  **power** | 1.00  (1.00,  1.00) | 1.00  (1.00,  1.00) | 1.00  (1.00,  1.00) | 1.00  (1.00,  1.00) | 1.00  (1.00,  1.00) | 1.00  (1.00,  1.00) | 1.00  (1.00,  1.00) | 0.50  (0.50,  0.50) | 0.71  (0.71,  0.71) | 0.38  (0.38,  0.38) | 0.00  (0.00,  0.00) |  |
| **ISCA**  **power** | 1.00  (1.00,  1.00) | 1.00  (1.00,  1.00) | 1.00  (1.00,  1.00) | 1.00  (1.00,  1.00) | 1.00  (1.00,  0.99) | 1.00  (1.00,  0.96) | 1.00  (1.00,  0.50) | 0.50  (0.50,  0.00) | 0.71  (0.71,  0.00) | 0.31  (0.31,  0.00) | 0.00  (0.00,  0.00) |  |
