# Supplementary material for: Relationship Estimation from Whole-Genome Sequence Data
Source: PLoS Genet. 2014 Jan 30;10(1):e1004144. doi: 10.1371/journal.pgen.1004144 (PMC3907355; doi:10.1371/journal.pgen.1004144)
Supplement: Table S2 — Consistency in spurious IBD regions detected by each method. We only considered spurious regions whose observed/expected ratio is larger than 4 and genetic longer is longer than 1 MB. (Numerical values in parentheses are results for comparing regions that are longer than 2 MB). Pairwise correlation between two methods was calculated by “Jaccard similarity coefficient”, the ratio of overlapped region length to total region length. CEU (A) refers to the 34 European control genomes described in the main text. CEU (B) is an additional sample of 20 unrelated Europeans. (DOCX) [file pgen.1004144.s012.docx]

| **GERMLINE** | **34 CEU (A)** | **8 ASI** | **4 MXL** | **20 CEU (B)** |
| --- | --- | --- | --- | --- |
| **34 CEU (A)** | 1 | 0.55 (0.54) | 0.35 (0.35) | 0.71 (0.70) |
| **8 ASI** |  | 1 | 0.38 (0.42) | 0.54 (0.56) |
| **4 MXL** |  |  | 1 | 0.42 (0.44) |
| **20 CEU (B)** |  |  |  | 1 |

| **fastIBD** | **34 CEU (A)** | **8 ASI** | **4 MXL** | **20 CEU (B)** |
| --- | --- | --- | --- | --- |
| **34 CEU** | 1 | 0.62 (0.68) | 0.36 (0.38) | 0.25 (0.30) |
| **8 ASI** |  | 1 | 0.27 (0.31) | 0.26 (0.25) |
| **4 MXL** |  |  | 1 | 0.34 (0.44) |
| **20 CEU** |  |  |  | 1 |

| **ISCA** | **34 CEU (A)** | **8 ASI** | **4 MXL** | **20 CEU (B)** |
| --- | --- | --- | --- | --- |
| **34 CEU (A)** | 1 | 0.36 (0.42) | 0.31 (0.38) | 0.58 (0.61) |
| **8 ASI** |  | 1 | 0.30 (0.38) | 0.37 (0.43) |
| **4 MXL** |  |  | 1 | 0.33 (0.39) |
| **20 CEU (B)** |  |  |  | 1 |
