# Supplementary material for: Relationship Estimation from Whole-Genome Sequence Data
Source: PLoS Genet. 2014 Jan 30;10(1):e1004144. doi: 10.1371/journal.pgen.1004144 (PMC3907355; doi:10.1371/journal.pgen.1004144)
Supplement: Table S3 — Excess IBD regions in Table 3 and their observed/expected ratio in the 34 European control genomes described in the main text (A) and 20 additional unrelated Europeans (B). (DOCX) [file pgen.1004144.s013.docx]

| **Chrom-osome** | **Starting position** | **Ending position** | **Genetic length**  **(in cM)** | **GERMLINE** | | **fastIBD** | | **ISCA** | |
| --- | --- | --- | --- | --- | --- | --- | --- | --- | --- |
|  |  |  |  | **A** | **B** | **A** | **B** | **A** | **B** |
| **chr9** | **38,293,483** | **72,605,261** | 8.15 | 39 | 16 | 10 | 11 | 2 | 0.4 |
| **chr8** | **10,428,647** | **13,469,693** | 7.96 | 38 | 14 | 2 | 5 | 4 | 5 |
| **chr21** | **16,344,186** | **19,375,168** | 6.91 | 22 | 8 | 2 | 2 | 0 | 0.7 |
| **chr10** | **44,555,093** | **53,240,188** | 7.58 | 22 | 13 | 2 | 0.8 | 3 | 3 |
| **chr22** | **16,051,881** | **25,095,451** | 20.82 | 22 | 12 | 3 | 2 | 6 | 3 |
| **chr2** | **85,304,243** | **99,558,013** | 6.53 | 21 | 11 | 2 | 0.7 | 1 | 0.2 |
| **chr1** | **118,434,520** | **153,401,108** | 9.95 | 19 | 18 | 47 | 48 | 811 | 69 |
| **chr15** | **20,060,673** | **25,145,260** | 10.46 | 15 | 7 | 42 | 33 | 0 | 1.3 |
| **chr17** | **77,186,666** | **78,417,478** | 5.66 | 11 | 4 | 0.1 | 0.6 | 0 | 1.8 |
| **chr15** | **27,115,823** | **30,295,750** | 9.29 | 9 | 7 | 3 | 1.2 | 0 | 1.6 |
| **chr17** | **59,518,083** | **64,970,531** | 6.23 | 9 | 5 | 4 | 2 | 0 | 0 |
| **chr2** | **132,695,025** | **141,442,636** | 9.16 | 7 | 3 | 4 | 2 | 0 | 3 |
| **chr16** | **19,393,068** | **24,031,556** | 6.18 | 6 | 4 | 5 | 5 | 0 | 0.3 |
| **chr2** | **192,352,906** | **198,110,229** | 5.04 | 4 | 4 | 4 | 1.5 | 0 | 0 |
| **Total** | **14 regions** |  | 119.92 |  |  |  |  |  |  |
