# Supplementary material for: Relationship Estimation from Whole-Genome Sequence Data
Source: PLoS Genet. 2014 Jan 30;10(1):e1004144. doi: 10.1371/journal.pgen.1004144 (PMC3907355; doi:10.1371/journal.pgen.1004144)
Supplement: Table S4 — Comparison regions identified in Table 3 with regions influenced by positive selection [16]. (DOCX) [file pgen.1004144.s014.docx]

| **Regions corresponding to the top signal of IBD sharing for each population** [[16](file:///C:\Users\hong\Downloads\ERSA%20response%2020131127.docx#_ENREF_16)] | | | **Excess IBD region in Table 3** |
| --- | --- | --- | --- |
| **Chromosome** | **Position (Mb)** | **Populations** |  |
| chr1 | 56.0 | CHD |  |
| chr2 | 50.9–51.2 | GIH, YRI, CHD |  |
| chr2 | 52.55 | LWK |  |
| chr3 | 84.9–85.3 | JPT, CHB |  |
| chr5 | 153.2–153.8 | MEX, CEPH |  |
| chr6 | 29.3–31.5, (w/o MKK 31.2–31.5) | All populations |  |
| chr8 | 8.4–10.5 | CEPH, CHB, CHD, GIH, JPT, MEX, TSI | chr8: 10,428,647-13,469,693 |
| chr11 | 49.3–51.1 | CEPH, CHD, JPT, MEX |  |
| chr11 | 55.2–56.7 | TSI, MKK, YRI |  |
| chr14 | 77.7 | ASW |  |
