# Supplementary material for: Relationship Estimation from Whole-Genome Sequence Data
Source: PLoS Genet. 2014 Jan 30;10(1):e1004144. doi: 10.1371/journal.pgen.1004144 (PMC3907355; doi:10.1371/journal.pgen.1004144)
Supplement: Table S5 — Comparison regions identified in Table 3 with long-range haplotypes reported by Gusev et. al. [17]. (DOCX) [file pgen.1004144.s015.docx]

| **Annotated regions enriched for IBD sharing between HapMap Populations** [[17](file:///C:\Users\hong\Downloads\ERSA%20response%2020131127.docx#_ENREF_17)] | **Excess IBD region in Table 3** | |
| --- | --- | --- |
| chr4 p15.1-p14 (27,700,000-41,200,000) |  |  |
| chr8 p23.1-p22 (6,200,000-19,000,000) | chr8: 10,428,647-13,469,693 |  |
| chr1 p36.21-p36.13 (12,700,000-20,400,000) |  |  |
| chr17 q22-q23.2 (502,000,00-61,100,000) |  |  |
| chr2 q32.3-q33.1 (191,900,000-203,300,000) | chr2:192,352,906-198,110,229 |  |
| chr16 p13.11-p12.3 (14,800,000-21,200,000) | chr16: 19,393,068-24,031,556 |  |
| chr21 q21.1 (16,400,000-24,000,000) | chr21:16,344,186-19,375,168 |  |
| chr9 p24.3 (0-2,200,000) |  |  |
| chr2 p25.1 (7,100,000-12,200,000) |  |  |
| chr1 p34.3 (34,600,000-40,100,000) |  |  |
| chr1 q42.3-q43 (234,700,000-243,700,000) |  |  |
| chr5 q23.1 (115,200,000-121,400,000) |  |  |
| chr4 q32.1 (155,600,000-161,800,000) |  |  |
| chr4 q32.3-q33 (164,500,000-171,900,000) |  |  |
| chr22 q11.22-q11.23 (22,200,000-23,500,000) | chr22: 16,051,881-25,095,451 |  |
| chr10 q21.1 (52,900,000-61,200,000) |  |  |
