# Supplementary material for: Relationship Estimation from Whole-Genome Sequence Data
Source: PLoS Genet. 2014 Jan 30;10(1):e1004144. doi: 10.1371/journal.pgen.1004144 (PMC3907355; doi:10.1371/journal.pgen.1004144)
Supplement: Table S6 — Comparison regions identified in Table 3 with long-range haplotypes reported by Price et. al. [18]. (DOCX) [file pgen.1004144.s016.docx]

| **Long-range LD regions** [[18](file:///C:\Users\hong\Downloads\ERSA%20response%2020131127.docx#_ENREF_18)] | **Excess IBD region in Table 3** |
| --- | --- |
| chr1: 48–52 Mb |  |
| chr2: 86–100.5 Mb | chr2:85,304,243-99,558,013 |
| chr2: 134.5–138 Mb | chr2: 132,695,025-141,442,636 |
| chr2: 183–190 Mb |  |
| chr3: 47.5–50 Mb |  |
| chr3: 83.5–87 Mb |  |
| chr3: 89–97.5 Mb |  |
| chr5: 44.5–50.5 Mb |  |
| chr5: 98–100.5 Mb |  |
| chr5: 129–132 Mb |  |
| chr5: 135.5–138.5 Mb |  |
| chr6: 25.5–33.5 Mb |  |
| chr6: 57–64 Mb |  |
| chr6: 140–142.5 Mb |  |
| chr7: 55–66 Mb |  |
| chr8: 8–12 Mb | chr8: 10,428,647-13,469,693 |
| chr8: 43–50 Mb |  |
| chr8: 112–115 Mb |  |
| chr10: 37–43 Mb |  |
| chr11: 46–57 Mb |  |
| chr11: 87.5–90.5 Mb |  |
| chr12: 33–40 Mb |  |
| chr12: 109.5–112 Mb |  |
| chr20: 32–34.5 Mb |  |
